# Rare dentin defects: Understanding the pathophysiological mechanisms of *COLXVA1* mutations

**DOI:** 10.1016/j.gendis.2024.101303

**Published:** 2024-04-20

**Authors:** Isaac Maximiliano Bugueno, Tristan Rey, Alexandra Jimenez-Armijo, Marzena Kawczynski, Naji Kharouf, Marie-Cécile Manière, Yann Herault, Agnès Bloch-Zupan, Virginie Haushalter-Laugel

**Affiliations:** a Institute of Genetics and Molecular and Cellular Biology (IGBMC), CNRS- UMR7104, INSERM U1258, Université de Strasbourg, Illkirch 67400, France; b Faculty of Dentistry, Université de Strasbourg, Strasbourg 67000, France; c Institute of Advanced Studies (USIAS), Université de Strasbourg, Strasbourg 67045, France; d Reference Center for Rare Oral and Dental Diseases (CRMR-O-Rares), Oral Medicine and Surgery Department, Hôpitaux Universitaires de Strasbourg (HUS), Strasbourg 67000, France; e Rare Diseases Health Network “TETE COU” & European Reference Network ERN CRANIO, Strasbourg 67000, France; f “Institut de Génétique Médicale d'Alsace”, Laboratory of Genetic Diagnostic, Hôpitaux Universitaires de Strasbourg (HUS), Strasbourg 67000, France; g Biomaterials and Bioengineering Laboratory, Inserm UMR_S 1121, Strasbourg 67000, France; h Orofacial Development and Regeneration, Faculty of Medicine, Institute of Oral Biology, Centre of Dental Medicine, University of Zürich, Zürich 8032, Switzerland

Dentin is a mineralized tissue with a chemical composition similar to bone but with a higher mineralized density and rigidity. It constitutes the central structure of the tooth between the internal pulp and external enamel toward the oral cavity or cementum toward the underlying roots. Inherited dentin defects occur in a variety of rare genetic diseases. They can manifest as “isolated” occurrences such as in dentinogenesis imperfecta (DI) or dentin dysplasia (DD) or can be associated with other symptoms in diseases such as osteogenesis imperfecta, Goldblatt syndrome, microcephalic osteodysplastic primordial dwarfism type II, among others.^1^

DI and DD are characterized by an abnormal formation and thus an abnormal structure of the dentin. There are classically three types of DI [DI-I (associated with osteogenesis imperfecta), DI-II, and DI-III], and two types of DD (DD-I and DD-II) according to the classification of Shields (1973) essentially based on phenotypic aspects and amber colored teeth. Patients with rare dentin defects are often misdiagnosed due to a complex phenotypic classification and limited knowledge of the genetic mechanisms involved. Currently, these defects have been linked to autosomal dominant inheritance of a handful of genes, namely *DSPP* (several variants of this gene are associated with different phenotypes of DI), *COLIA1*, *COLIA2*, *COLIIIA1*, and others. *DSPP* gene, being involved in DI-II, DI-III, and DD-II, has been presumed to be the mutated factor in all isolated forms of DI presenting a variable expressivity from mild to severe according to a classification proposed in 2015.^2^ For syndromic DI, mutations in the genes coding for collagen I proteins, *COLIA1* and *COLIA2*, or for collagen-modifying enzymes and chaperone proteins (*CRTAP*, *LEPRE1*, *PPIB*, *FKBP10*, *SERPINH1)* can also participate in disease etiology. In dentin dysplasia type I (short roots), only 3 genes have been described to date, *SMOC2*, *SSUH2*, and *VPS4B*. Genetic mutations causing DI-I are often linked to the formation of collagen triple helixes, thus impacting the pathophysiology of osteogenesis or dentinogenesis. Several other collagen genes have been described as important for bone and dentin morphogenesis and homeostasis.^3^ Among them, the collagen type XVa1 gene (*COLXVA1*) participates in both bone and tooth metabolism. This collagen displays enhanced expression in new-forming matrix osteoblasts and is strongly increased during osteogenic differentiation. As such, COLXV is secreted by odontoblasts in the cell matrix of the newly formed dentin (pre-dentin).

Here, we examined related individuals with a DD-I phenotype with autosomal dominant transmission. The phenotype (Fig. 1A; Fig. S2) was present over several generations as observed on the family tree (Fig. 1B). The main clinical features were hyperlaxity of joints, the dysmorphic sign on the feet hallux varus, and frostbite-like injuries. Affected individuals showed sensitivity and injuries when exposed to low temperatures, causing damage to the skin on fingers and toes, suggesting possible microcirculatory changes and aberrant angiogenesis. Regarding teeth, the roots were sharp with conical and apical constrictions. Pulpal obliteration led to a crescent-shaped pulpal remnant and total pulpal obliteration occurred in most of the teeth (Fig. 1C, D; Fig. S1, 3). Patients complained of painful teeth. Scanning electron microscopy was performed on avulsed teeth of affected members of the family, allowing analysis of enamel and dentin structural anomalies (Fig. 1D). While the number and size of dentinal tubules were normal, most of them were open and almost empty; and some tubules were closed and calcified (sclerosis) (Fig. 1C, D). The micrographs from scanning electron microscopy showed many abnormal calcified odontoblastic extensions with mineralized peri- or inter-tubular dentin but without collagen fibers. Some other tubules had destroyed calcified tips and demineralized collagen fibers in the peri- or inter-tubular dentin. A very thin enamel layer at the tooth cervical area was observed (Fig. 1C, D; Fig. S3). Hypocalcified lesions on enamel could be seen, but no major enamel defects were detected.

**Figure 1:**
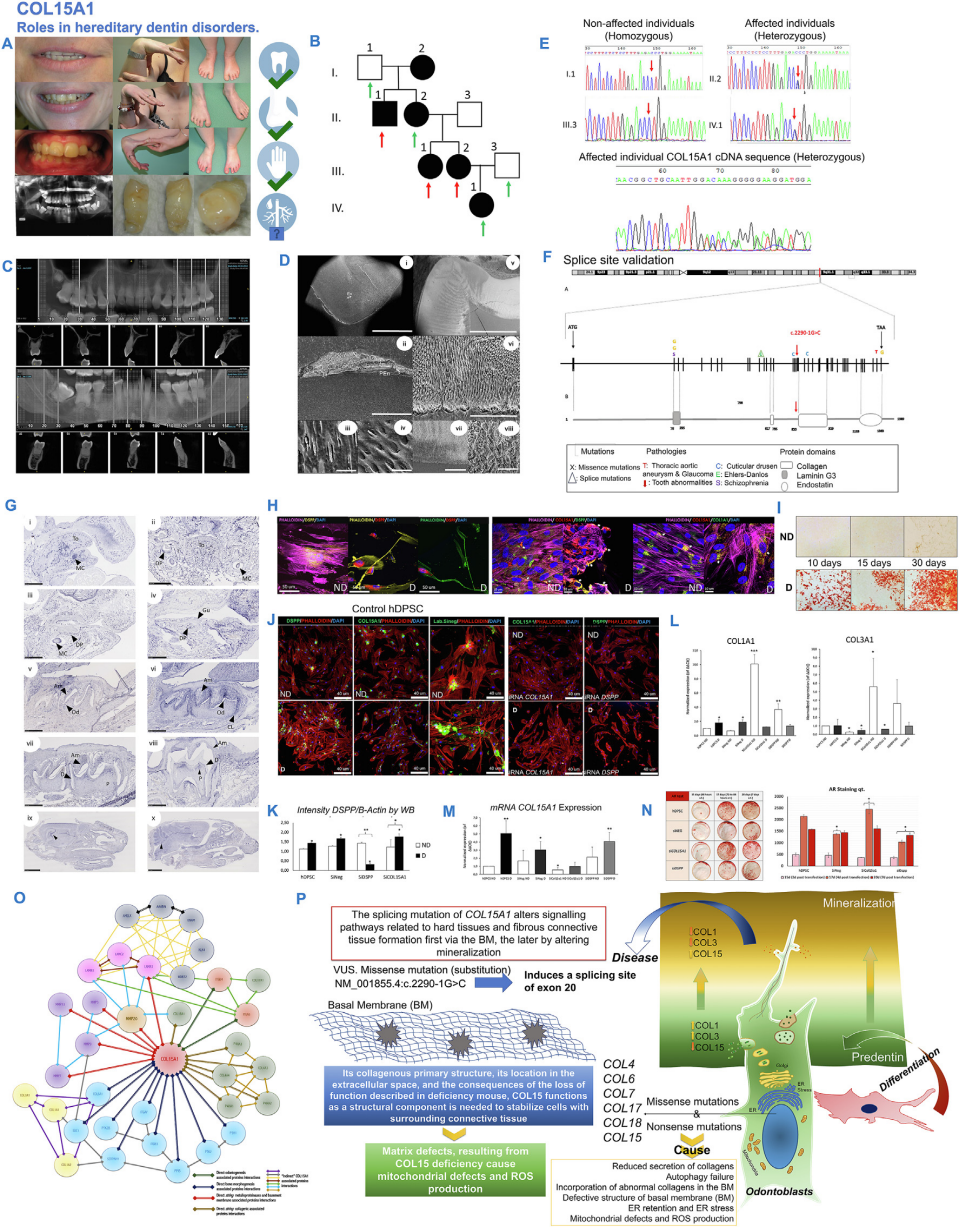
Understanding the pathophysiological mechanisms of *COLXVA1* mutations in rare dentin defects. **(A)** Clinical features of affected individuals. The red arrows indicate patients who underwent whole exome sequencing and the green arrows show patients who underwent family segregation by Sanger. **(B)** Pedigree of a family affected by a form of dentin dysplasia (rather type I) with autosomal dominant transmission **(C)** CBCT reconstruction in 3D showed clearly the obliteration of all pulp chambers with short, blunted, and malformed or absent roots. In CBCT images, the full upper and lower jaws are presented, and some sections of premolars, molars, and incisors are shown with the same morphological pattern. **(D)** (i) Dentin structural alterations on affected individual's teeth (SEM micrograph 30X magnification). Coronal dentin and the thin enamel layer at the tooth cervical area (En: Enamel). (ii) High magnification of the thin enamel layer. (iii) High magnification of the calcified odontoblastic extensions. (iv) Mineralized odontoblastic process and the demineralized collagen fibers in the peri- or inter-tubular dentin. (v) Enamel light structural alterations on affected individual's teeth (SEM micrograph 50 × and 70 × magnification). Coronal enamel with a light-altered pattern. (vi, vii) Higher magnification showed an altered dentin-enamel junction. (viii) High magnification on enamel prisms showed enamel focalized defects and impaired enamel prism structure. **(E)** Affected and non-affected family members' *COLXVA1* cDNA amplification. Non-affected members: I.1 and III.3. Affected members: II.2, III.1, and III.2. Sequencing of the amplification product of affected member II.2 showed two overlapping sequences. The first sequence corresponds to exon 19 followed by the exon 20 sequence. The second sequence corresponds to the exon 19 sequence followed by exon 21. This confirmed a heterozygous exon 20 skipping. **(F)** Representation of *COLXVA1* human gene located on chromosome 9. The mutation detected in this study is highlighted by a red arrow on intron 19 (NM_001855.4:c.2290-1G > C). Previously described mutations in the *COLXVA1* gene are symbolized by a single letter above the corresponding exon. **(G)** The selected sections illustrating *ColXVa1* expression features during mouse tooth development, specifically lower molars. (i) *ColXVa1* molar expression in the ectomesenchyme cells at embryonic day 13.5 (E13.5) (ii) and at E14.5 (iii) and in the dental papilla at E16.5 (iv); at post-natal day 1 (P1) (v), at P3 (vi), at P7 (vii), and at P14 (viii). (ix) Hippocampus expression. (×) Cerebellar *ColXVa1* expression (black arrowheads). Am, ameloblasts; CL, cervical loop; D, dentin; DP, dental papilla; Gu, gubernaculum; MC, Meckel's cartilage; Od, odontoblasts; P, dental pulp; To, tongue. **(H)** Spinning disk confocal immunofluorescent microscopy for DSPP, COLXVA1, and COLA1A1 in non-differentiated hDPSCs (ND-hDPSCs) and differentiated hDPSCs (D-hDPSCs) into odontoblast like-cells. **(I)** Mineralization evaluation by alizarin red test for ND- and D-hDPSCs. **(J)** DSPP and COLXVA1 protein expression by immunofluorescence of ND- and D-hDPSCs transfected with siRNA. Magnification for all images was 40X and scale bars are represented on each capture. **(K)** The intensity ratio of DSPP on B-actin was plotted for ND- and D-hDPSCs without transfection, and transfected with siNEG, siDSPP, and siCOLXVA1. **(L)***COLIA1* and *COLIIIA1* mRNA expression by quantitative reverse-transcription PCR of ND- and D-hDPSCs transfected with siCOLXVA1 and siDSPP. **(M)***COLXVA1* mRNA expression by quantitative reverse-transcription PCR of ND- and D-hDPSC transfected with siCOLXVA1 and siDSPP. **(N)** Mineralization evaluation with alizarin red test for transfected cells after siRNA *COLXVA1* and *DSPP* transfection. Alizarin tests were performed at 3 (day 14 of differentiation), 6 (day 16 of differentiation), and 8 (day 18 of differentiation) days after transfection of D-hDPSCs with siNEG, SiCOLXVA1, and siDSPP. **(O)** Protein-protein interaction networks functional enrichment analysis constructed through STRING DATABASE. **(P)** Schematic figure of COLXVA1's role in odontoblast differentiation, mineralization, and its splicing mutation effect as a basement membrane structural component. CBCT, cone beam computed tomography; SEM, scanning electron microscopy; hDPSC, human dental pulp stem cell.

No variation in *DSPP* was found by targeted next-generation sequencing (GenoDENT panel) in any affected family member.^4^ To broaden the scope of analysis, we performed whole exome sequencing (Integragen) followed by read alignment. After a stringent exclusion (Supplemental Table S1) following clinical, genetic, and bioinformatic criteria, remarkably, a heterozygous mutation in the *COLXVA1* gene (NM_001855.4:c.2290-1G > C) inducing a splicing site of exon 20 was identified (Fig. 1E, F; Fig. S2). In addition, familial segregation was validated. Skipping of exon 20, which codes for a consensus sequence of collagen, was confirmed through RNA analysis of gingival cells derived from affected individuals of the family. COLXVA1 is a secreted non-fibrillar collagen abundant in the tissue basement membrane. It is expressed by osteoblasts forming a bone matrix and it is secreted by odontoblasts into the cell matrix of newly formed dentin (pre-dentin). *COLXVA1* gene expression was reported as strongly increased during osteogenic differentiation and during mineralization in human odontoblasts differentiated from dental pulp cells *in vitro*.^5^

To further study the role of this gene in dentinogenesis, analysis of mRNA *ColXVa1* expression and distribution during mouse dental development was performed by *in situ* hybridization. Elevated *ColXVa1* expression in mouse odontoblasts, dental pulp, and cerebellum was observed along tooth and head development of mice (Fig. 1G; Fig. S4). A functional evaluation through expression inhibition was performed by *in vitro* cell culture of human dental pulp stem cell differentiated into odontoblast-like cells, followed by siRNA transfection and subsequent quantitative real-time PCR analysis, Western blot, and high-speed multispectral spinning-disk confocal microscope system for immunofluorescence of cells. Odontoblast-like differentiated cells from human dental pulp stem cells express dentinogenesis-related proteins (MMP20, DSPP, and COLIA1) that co-localize with COLXVA1 (Fig. S6). Interestingly, COLXVA1 displayed a similar up-regulation pattern during odontoblast differentiation (Fig. 1H, I; Fig. S5). To better assess the role of COLXVA1 in dentinogenesis, we used a cellular model for *COLVXA1* loss-of-function by siRNA transduction. *DSPP* RNA interference was performed in parallel as a positive control and resulted in strong alterations of dentin mineralization with potential pathogenic overproduction of *COLIA1* and *COLIIIA1* (Fig. 1J–M). *COLXVA1* inhibition blocks mineralization and modulates dentinogenesis-related proteins during odontoblast differentiation from human dental pulp stem cells (Fig. 1J–N; Fig. S6). Quantitatively, fewer mineralization spots and coloration were observed with a slower recovery of normal mineralization over time, which more clearly stated a different behavior when comparing *COLXVA1* inhibition with *DSPP* inhibition (Fig. 1N). Finally, an interactome of COLXVA1 was performed building a full protein network (see supplementary materials and methods; Fig. 1O; Fig. S7, 8 and Table S3). Hypothetically COLXVA1 alterations may lead to impaired basement-membrane collagen structure (COL1A1, COL3A1), but also impaired metalloproteinase activity (via MMP20) with implications in both dentinogenesis and amelogenesis during tooth development (Fig. 1 H–N; Fig. S5–8).

These investigations support an emerging and growing role of COLXVA1 in osteogenesis and dentinogenesis involving *COLXVA1* in the molecular diagnosis of dentin dysplasia (type I) and creating a model deciphering newly characterized signaling pathways critical to normal dentin formation (Fig. 1O). *COLXVA1* expression is high during osteogenesis or odontogenesis induction and matrix secretion and is then decreased, once the calcified matrix is formed. Hypothetically, COLXVA1 could play a role in the regulation of hydroxyapatite matrix deposits within the extracellular matrix as mutations of other collagens affect bone mineralization. Potentially, COLXVA1 would not participate as a minor structural protein in the predentin matrix, but rather as an organizer establishing a mature dentin matrix and facilitating mineralization via hydroxyapatite deposits at the right time and place. Impaired expression of COLIA1 and COLIIIA1 (the main proteins of the non-mineralized matrix) could contribute to dentin defects. In fact, during osteogenic differentiation and consequent mineralization, a down-regulation of *COLIA1* and *RUNX2* (observed by RNA sequencing) was associated with an up-regulation of *COLXVA1*. We showed that *COLXVA1* siRNA knockdown, during odontoblast differentiation, produced ectopic overall mineralization. Combined with patient phenotypic data COLXVA1 clearly regulates the formation of the mineralized dentin (Fig. 1P).

Basement membrane components may play an important role in cell behavior and pre-mineralized matrix, such as predentin. Mutations affecting genes coding for basement membrane components, such as laminin, collagen IV, or odontogenic ameloblast-associated protein, can directly affect odontogenesis. Numerous mutations in basement membrane collagen genes are associated with a wide variety of genetic diseases. Endoplasmic reticulum retention and endoplasmic reticulum stress or autophagy induction, represent potential convergent disease mechanisms for several collagen malfunctions and basement membrane defects. This again supports our postulate of the increasing roles and importance of identified mutations in this collagen XV (here a splicing site at the terminus of exon 19 and upstream of the collagen domain of this gene inducing exon 20 skipping) and how broad the associated phenotypic spectra could be. Collectively, the data appear to confirm this *COLXVA1* variant as likely pathogenic and responsible for the DD-I phenotype encountered in this family.

## Ethics declaration

The patients' oral phenotype was documented using the D [4]/phenodent registry protocol. This clinical study is registered at https://clinicaltrials.gov: NCT01746121/NCT02397824 and with the French Ministry of Higher Education and Research Bioethics Commission as a biological collection “Orodental Manifestations of Rare Diseases” DC-2012-1677/DC-2012-1002; it was acknowledged by the person protection committee. The parents gave written informed consents for the genetic analyses performed on the salivary samples (OG-250 Oragene®DNA kit, DNA Genotek Inc., Ottawa, Ont., Canada, www.dnagenotek.com) both for them and their children in accordance with the Declaration of Helsinki. They also gave written consent participating to D [4]/phenodent registry and to the publication of this article and the clinical images that are presented**.** A small piece of gingiva was also taken during a programmed surgery procedure from patient II.2 to extract RNA and confirm exon 20 skipping.

The animal experiments were performed in accordance with the French national and European Laws and Directives Concerning Laboratory Animal Housing, Welfare, and Experimentation and after approval from the CERBM-GIE: ICS/IGBMC Ethical Research Board.

## Funding

This work was financed by and contributed to the actions of the projects Offensives Sciences INTERREG IV A27 and “RARENET: No. 1.7, a trinational network for education, research and management of complex and rare disorders in the Upper Rhine” co-financed by the European Regional Development Fund (ERDF) of the European Union in the framework of the INTERREG V Upper Rhine program as well as to the ERN (European reference network) CRANIO initiative. ABZ is a USIAS 2015 Fellow of the Institute of Advanced Studies (Institut d’Etudes Avancées) de l’Université de Strasbourg, France. It was also supported by the grant ANR-10-LABX-0030-INRT, a French State fund managed by the Agence Nationale de la Recherche under the frame programme Investissements d’Avenir labelled ANR-10-IDEX-0002-02. This work is also part of the Projet E-GENODENT financed by the Fonds d’Intervention Régionale (FIR) of the Agence Régionale de Santé Grand Est (2019-2022). The authors thank the “Impulsion Recherche” financial support of the “Filière de Santé Maladies Rares TETECOU” 2021 and 2022 and acknowledge the Fondation Force for supporting the DIAGNODENT project (2023-2026). The funders had no role in the study design, data collection and analysis, decision to publish, or preparation of the manuscript.

## Conflict of interests

The authors declared no conflict of interests in this study.
