# Supplementary material for: Rare dentin defects: Understanding the pathophysiological mechanisms of COLXVA1 mutations
Source: Genes Dis. 2024 Apr 20;11(5):101303. doi: 10.1016/j.gendis.2024.101303 (PMC11074959; doi:10.1016/j.gendis.2024.101303)
Supplement: Multimedia component 1 [file mmc1.pdf]

<u>*Rapid communication supplemental data*</u>

Title: Rare dentin defects: understanding the pathophysiological mechanisms of COLXVA1 mutations.

A dentin dysplasia type-I phenotype was clinically diagnosed on affected family members

Several members of the described family were affected by DD-I with autosomal dominant transmission. The phenotype was present over several generations as observed on the family tree (**Sup. Fig. 1A**). The main clinical features were hyperlaxity of joints, the dysmorphic sign on the feet "*hallux varus*" and frostbite-like injuries. Affected individuals showed sensitivity and injuries when exposed to low temperatures, causing damage to the skin on fingers and toes (**Sup. Fig. 1B**), suggesting possible microcirculatory changes and aberrant angiogenesis **(1,2)**. Regarding teeth, the roots were sharp with conical and apical constrictions. Pulpal obliteration occurs leading to a crescent-shaped pulpal remnant and total pulpal obliteration in most of the teeth (**Sup. Fig. 1 and 3**). These features were confirmed by CBCT 3D analysis and Micro-CT scanning of an extracted tooth (**Fig.1 and Sup. Fig. 3**). Radiographically, the phenotype manifests as obliteration of all pulp chambers, short, blunted, and malformed or absent roots with periapical lesions involving apparently intact teeth (**Sup. Fig. 1B**). Patients complained of painful teeth.

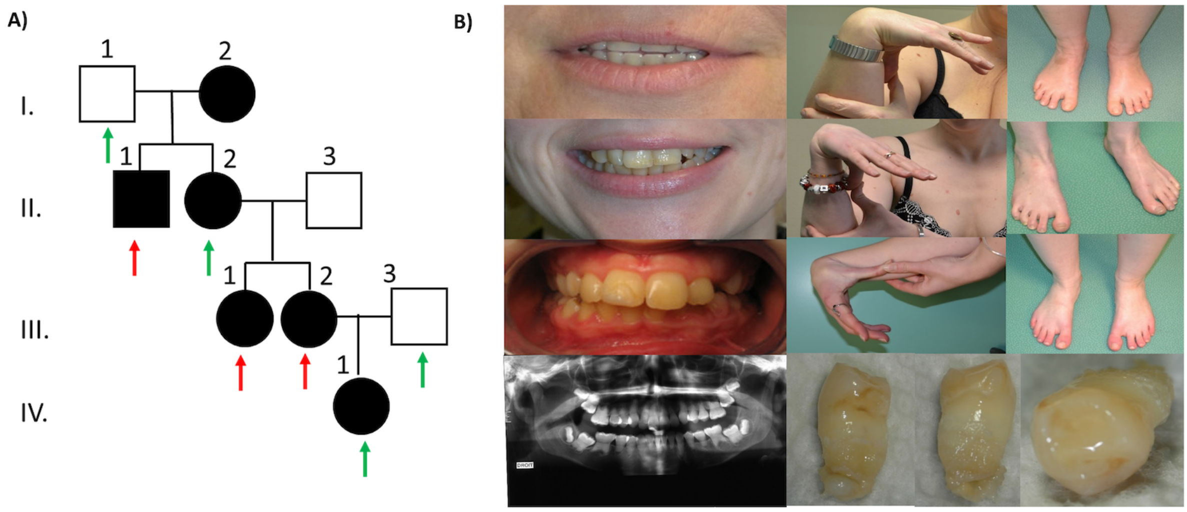

Supplemental figure 1. Clinical features and mode of inheritance of dentin dysplasia type I in a French family. *A. Pedigree of a family affected by a form of dentin dysplasia (rather type I) with autosomal dominant transmission.* ***B.*** *Clinical features of affected individuals: hyperlaxity of joints, dysmorphic sign on the feet hallux varus, frostbite, teeth roots are sharp with conical and apical constrictions, a crescent-shaped pulpal remnant and total pulpal obliteration in most of teeth, obliteration*

of all pulp chambers, short, blunted, and malformed or absent roots with periapical radiolucency involving apparently intact tooth. Red arrows indicate patients underwent Whole Exome Sequencing and green arrows show patients underwent Family Segregation by Sanger.

A heterozygous mutation in the COLXVA1 gene (NM_001855.4:c.2290-1G>C) was identified and confirmed in affected individuals and collected cells.

In order to determine the inherited mutation, targeted next-generation sequencing was performed on salivary samples of the family and no mutations in candidate genes within the GenoDENT panel were identified **(3,4)**. To broaden the scope of analysis, we performed Whole Exome Sequencing (WES; Integragen) followed by read alignment.

Structural Variations' annotation was performed and no candidate was retained (data not shown). Other variants were excluded based on high allele frequency, by their position and protein effect prediction, and for lacking autosomal dominant transmission. 53 heterozygous variants in 53 different genes were left for a manual curation (review of literature, animal models, description in other diseases, and in the strength of the mutation) (**Sup. Fig. 1B, 2A-C and Sup. Table 1**). After this stringent exclusion, two heterozygous variants were kept. One in *COLIIIA1* NM_000090.3:c.3937A>C; p.(Lys1313Gln) and one in *COLXVA1* NM_001855.4:c.2290-1G>C. The variant in *COLIIIA1* is predicted to change a well-conserved Lysine into a Glutamine and the computational tools SIFT (v6.2.0) and MutationTaster (v2021) predicted the variant's impact as deleterious. This variant has been sequenced 3 times in the gnomAD database but never as homozygous mutation. Additionally, it has only be reported as variant of uncertain significance (VUS) and never as class IV or V (likely pathogenic or pathogenic variant). As the *COLIII1* gene is involved in Ehlers-Danlos vascular type (OMIM# 130050) we referred our patients for cardiovascular examination, but no cardiovascular anomalies were diagnosed. This absence of Ehlers-Danlos-like cardiovascular anomalies reinforced our hypothesis that *COLIIIA1* was not the pathogenic variant in these patients.

The remaining VUS is within the *COLXVA1* gene, namely a heterozygous mutation (NM_001855.4:c.2290-1G>C)(**Sup. Fig. 1B, 2A-C**). This variant impacts the splicing site of exon 20. Indeed, the skipping of exon 20, which codes for a consensus sequence of collagen, was confirmed through RNA analysis of gingival cells derived from individuals of the family, (**Sup. Fig. 2**). In recent papers, this gene was reported as strongly increased during osteogenic differentiation **(5,6)**. COLXVA1 is a secreted non-fibrillar collagen abundant in the basement

membrane of tissues. It is expressed by osteoblasts forming bone matrix and it is secreted by odontoblasts into the cell matrix of newly formed dentin (pre-dentin) (6,7). Recent studies have shown that *COLXVA1* mRNA expression is increased during mineralization tests in human odontoblasts **(8)**, and COLXVA1 is increased during mineralization process in dental pulp cells *in vitro* **(7,8)**. In addition, familial segregation was validated. Collectively, the data appear to confirm this *COLXVA1* variant as pathogenic.

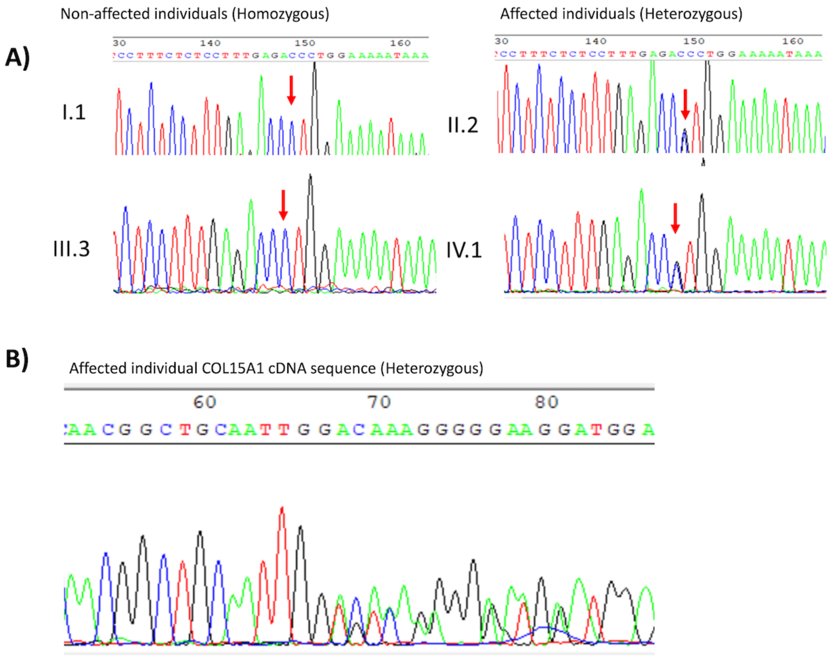

C)

VariantID	Gene	GeneDesc	OmimId	Mutation (VUS)	Zygosity	VarType	VarLocation	Assembly	Exon	Intron
9_101798451_G_C	COL15A1	Collagen, type XV, alpha 1	120325	NM_001855.4:c.2290-1G>C	het	substitution	splice site	GRCh37	20	19

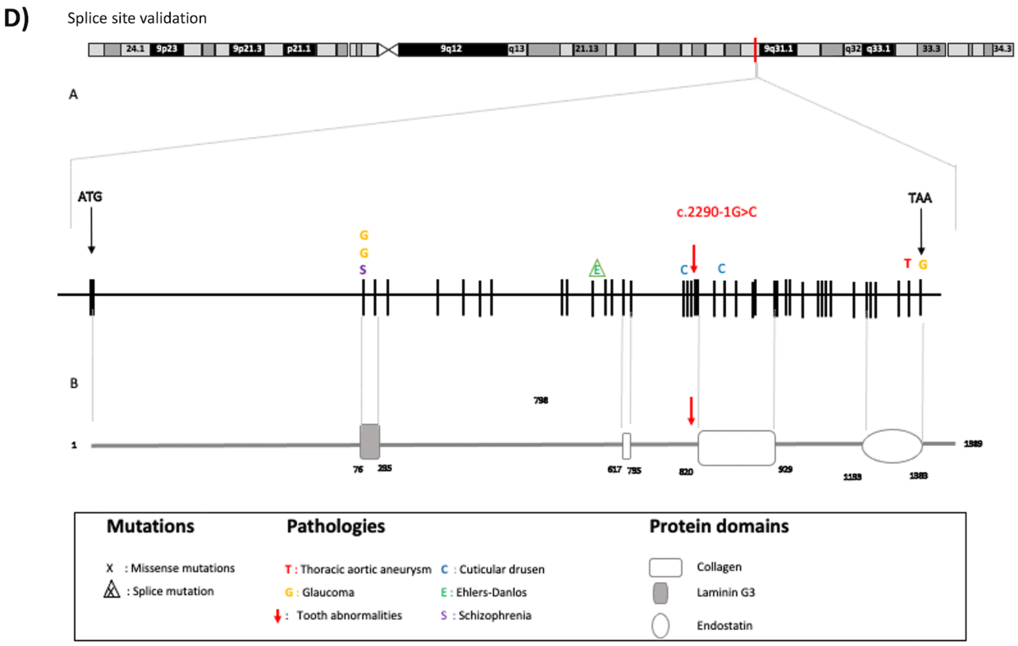

Supplemental figure 2. A heterozygous mutation in the COL15A1 gene (NM_001855.4:c.2290-1G>C) was identified in affected individuals. A. *Affected and non-affected family members' COLXVA1 cDNA amplification. Non-affected members (I.1, III.3) showed only one amplification band and appeared homozygous. Affected member's (II.2, III.1, III.2) showed two amplification bands and appeared heterozygous.* ***B.*** *Sequencing of the amplification product of affected member II.2 showed two overlapping sequences. The first sequence corresponds to exon 19 followed by exon 20 sequence. The second sequence correspond to exon 19 sequence followed by exon 21. This confirmed a heterozygous exon 20 skipping.* ***C.*** *Synthesis table of identified VUS in COLXVA1.* ***D.*** *Representation of COLXVA1 human gene located on chromosome 9. It contains 42 exons (each vertical black line denotes one exon). The position of the start codon (ATG) is indicated. The mutation detected in this study is highlighted by a red arrow on intron 19 (NM_001855.4:c.2290-1G>C). Previously described mutations in the COLXVA1 gene are symbolized by a single letter above the corresponding exon. The corresponding protein domains are represented according to the PFAM database.*

Tooth structure of affected individuals showed an alteration in dentin and enamel mineralization.

Scanning electron microscopy was performed on avulsed teeth of affected members of the family, allowing analysis of enamel and dentin structural anomalies. Patient phenotypes were very peculiar, showing ultrastructural closed and calcified dentinal tubules with mineralization of the odontoblastic processes. While the number and size of dentinal tubules were normal, most of them were open and almost empty; but some tubules were closed and calcified (sclerosis). It was possible to observe odontoblastic extensions on normal tubules (**Sup. Fig. 3A**). SEM micrograph showed many abnormal calcified odontoblastic extensions with mineralized peri- or inter-tubular dentin but without collagen fibers. Some other tubules had destroyed calcified tips and demineralized collagen fibers in the peri- or inter-tubular dentin (**Fig. 3A**). Enamel structure and its mineralization seemed highly affected (**Sup. Fig. 3B**). A very thin enamel layer at tooth cervical area was observed. At high magnification this layer showed aprismatic enamel was covered by a mineralized structure (dental calculus) (**Sup. Fig. 3A, 3B**). Hypocalcified lesions on enamel could be observed, but no major enamel defects were observed. However, high magnification electron microscopy analysis of teeth of affected patients showed definite defects in mineralization and organization of the dentin, which are indeed correlated with a diagnosis of dentin dysplasia (**Sup. Fig. 1C, 3A, 3B**).

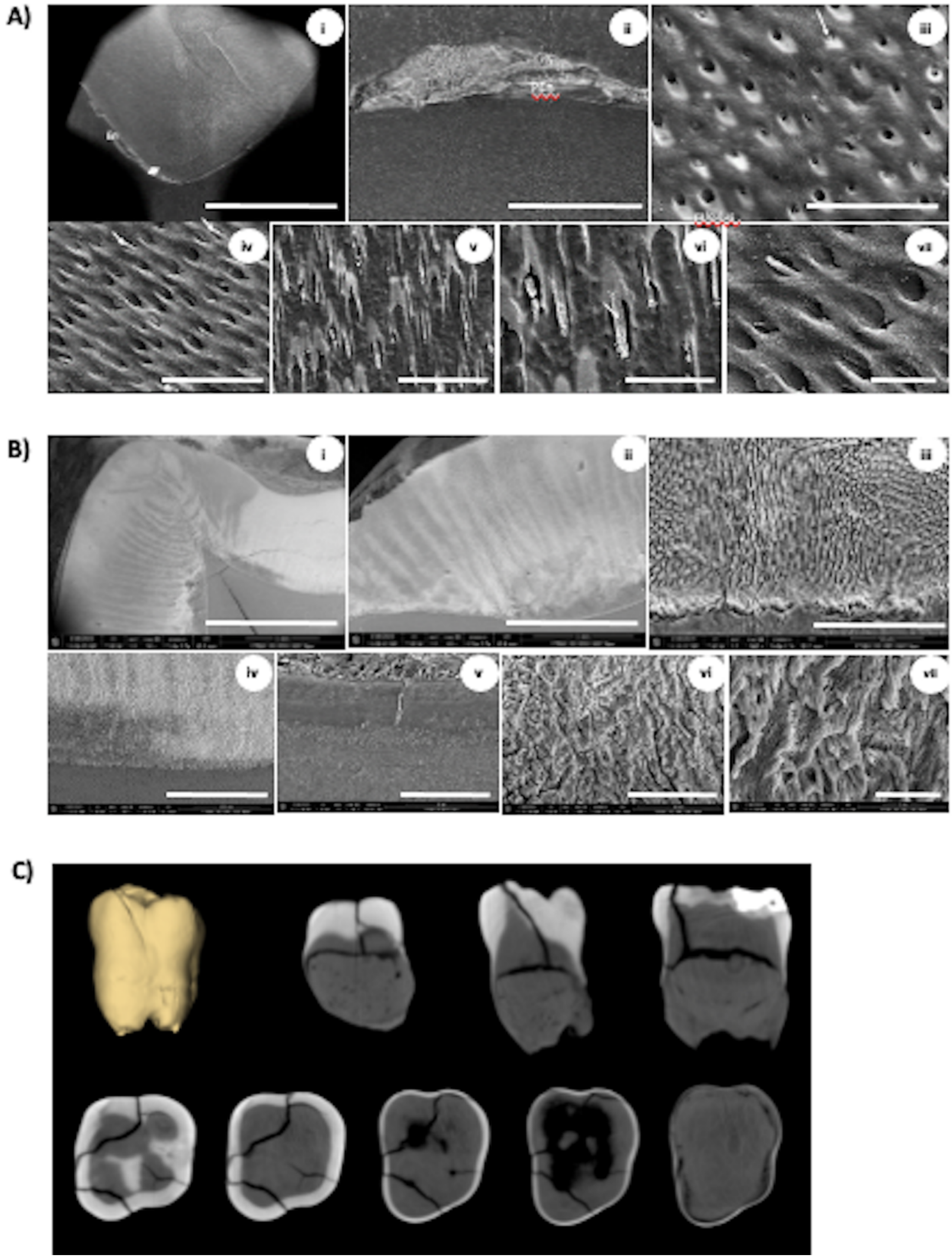

Supplemental figure 3. Dentin and enamel structure alterations on patient's teeth by scanning electron microscopy (SEM). A. *Dentin structural alterations on affected individual's teeth* ***(i)*** *SEM Micrograph 30X magnification. Coronal dentin showed no trace of the dental pulp. A very thin enamel layer at tooth cervical area (En: Enamel);* ***(ii)*** *High magnification of the thin enamel layer showed the aprismatic enamel covered by mineralized structure (probably calculus);* ***(iii)*** *SEM micrograph 3000X magnification. The dentinal tubules are open and almost empty with no odontoblastic processes. Some dentinal tubules are closed (probably sclerotic) (white arrow);* ***(iv)*** *High magnification of dentinal tubules showed some odontoblastic extensions (white arrows);* ***(v)*** *Numerous calcified odontoblastic*

processes with mineralized peri- or inter-tubular dentin but without collagen fibers; (**vi**) High magnification of the calcified odontoblastic extensions showed some destroyed calcified tips (white arrow). ***(vii)*** Mineralized odontoblastic process and the demineralized collagen fibers in the peri- or inter-tubular dentin. Dentinal tubules displayed different patterns according to the observed area ***B.*** *enamel light structural alterations on affected individual's teeth* ***(i, ii)*** *SEM Micrograph 50X and 70X magnification. Coronal enamel with a light altered pattern;* ***(iii, iv, v)*** *Higher magnification showed an altered dentin-enamel junction;* ***(vi, vii)*** *High magnification on enamel prisms showed enamel focalized defects and impaired enamel prism structure. C. Micro-CT scanning of an extracted lower permanent 2*[nd] *molar (tooth 47) of an affected family member showing the root shape and the obliteration or absence of root canals.*

Analysis of mRNA *ColXVa1* expression and distribution by *in situ* hybridization on mouse tissue

In situ hybridization for *ColXVa1* was performed on mice tissues, to evaluate mRNA expression and transcript distribution during mouse dental development. Elevated *ColXVa1* expression in mouse odontoblasts, dental pulp, and cerebellum was observed (**Sup. Fig. 4i-x**). *ColXVa1* molar expression began at E13.5 in bud stage ectomesenchyme cells. Elevated *ColXVa1* expression continued at E14.5 (cap stage) and E16.5 (bell stage) in the dental papilla. At post-natal day PN1 *ColXVa1* expression appeared to be localized to the pulp. At PN3, *ColXVa1* mRNA was also present in ameloblasts and odontoblasts. At P7 and P14, an enriched expression in pulp cusps (the most differentiated mesenchymal area) was observed (**Sup. Fig. 4**).

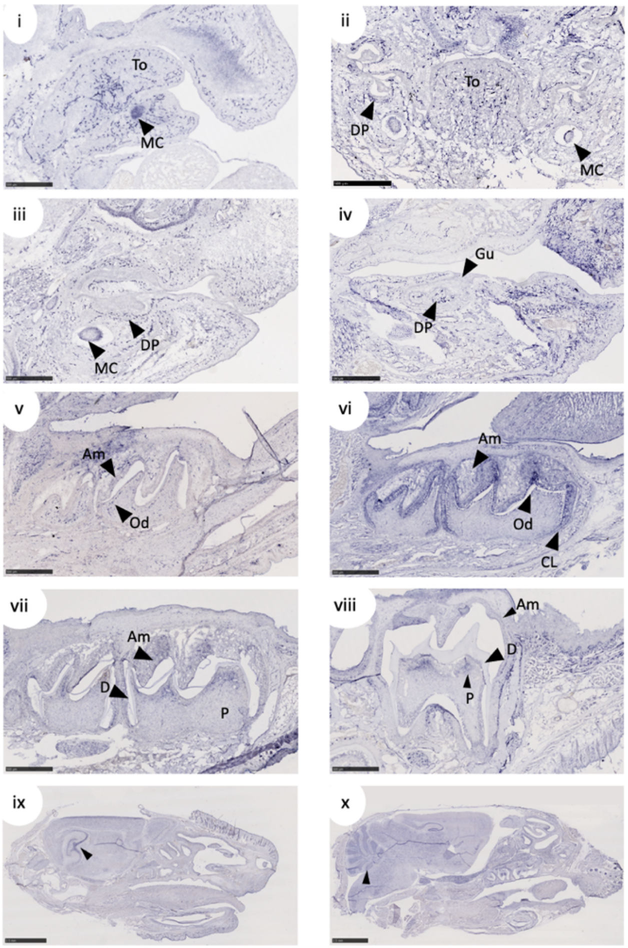

Supplemental figure 4. Analysis of mouse ColXVa1 transcripts distribution by in situ hybridization. *Selected sections illustrating Col15a1 expression features during mouse tooth development, specifically lower molars. (i,ii) Col15a1 molar expression begins at E13.5 in the ectomesenchyme cells, (iii) continues at E14.5 and (iv) E16.5 in the dental papilla. (v) At post-natal day PN1 appears to be localized in in dental pulp on several spots closed to odontoblasts. (vi) At PN3, Col15a1 mRNA expression was localized in odontoblasts. Ameloblasts and cervical loop showed strong Col15a1 expression. (vii)*

At P7 and (viii) P14, an enriched expression in pulp cusps is observed. (ix) Hippocampus and (x) cerebellar Col15a1 expression (black arrowheads). MC, Meckel's cartilage; To, tongue; DP, dental papilla; Gu, gubernaculum; Am, ameloblasts; Od, odontoblasts; CL, cervical loop; D, dentin; P, dental pulp. Scale bars: 500 µm

Odontoblast-like differentiated cells express dentinogenesis-related proteins which co-localize with COLXVA1

Morphological differences between human Dental Pulp Stem Cells (hDPSC) (**Sup. Fig. 5A-I**) and odontoblast-like cells were highlighted by immunochemistry and epi-fluorescence analysis of cells before and after differentiation for 21 days or more. hDPSC before differentiation show a flatter and spindle shape close to a typical ecto-mesenchymal morphology. Odontoblast-like cells differentiated from hDPSC after 21 days, were more compact with (in some cases) an odontoblastic-like extension of the cytoplasm. This feature was observed using a differentiation medium from 14 to 30 days (**Sup. Fig. 5A, 6A, B).** DSPP, MMP20, and COL1A1 are key proteins in odontogenesis and in particular dentinogenesis, which are collectively expressed in hDPSC (**Sup. Fig. 5A, C, D, 6A, B**). To assess the mineralizing functions of differentiated cells, the Alizarin Red test confirmed the presence of calcium deposits after 14 days of culture. A significant quantitative increase of red staining was visible throughout time during hDPSC grown in differentiation medium (**Sup. Fig. 5B, 6A, B**). The differentiation medium allowed cells to mature into odontoblast-like cells, hence displaying the ability to produce mineralized ECM overtime. At 30 days of culture, the highest presence of surface calcium deposits was observed (**Sup. Fig. 5B**). No specific staining was observed in non-differentiated cells (**Sup. Fig. 5B**). Immunofluorescence in non-differentiated (ND-) and differentiated (D-) hDPSC was performed to evaluate the expression of proteins involved in dentinogenesis (MMP20, DSPP, COL1A1 and COLXVA1) (**Sup. Fig. 5C, 5D**). All these proteins were expressed in ND- and D-hDPSC, but their expression level increased in D-hDPSC at 21 days of differentiation. A decentralization of the nucleus in a rectangle shaped cell was observed, mimicking the polarized shape of differentiated odontoblast final organization (**Sup. Fig. 5C, 5D**). D-hDPSC demonstrate higher expression of dentinogenesis proteins (MMP20, DSPP, and COLIA1) than ND-hDPSC (**Sup. Fig. 5D**). Interestingly, COLXVA1 displayed a similar upregulation (**Sup. Fig. 5D, 6A, B**). This confirmed that differentiation medium allowed hDPSC to mature into odontoblast-like cells with characteristic protein expression profiles after 21 days, strenghthening the utility of this cell line to investigate dentinogenesis *in vitro* (**Sup. Fig. 5A-E**). To measure

relative mRNA expression, RTqPCR on D- and ND-hDPSC was performed (**Fig. 5E**). D-hDPSC expressed more *COLXVA1* (Fold-change: 3.6 p<0,05) and *DSPP* (Fold-change: 1.77 p<0,05) mRNA than did ND-hDPCS consistent with their upregulated protein levels.. Notably, increased COLXVA1 and DSPP levels correlates with increased and progressive mineralization within the ECM in differentiated hDPSC after 21 days **(Sup. Fig. 5A-E, 6A, B).**

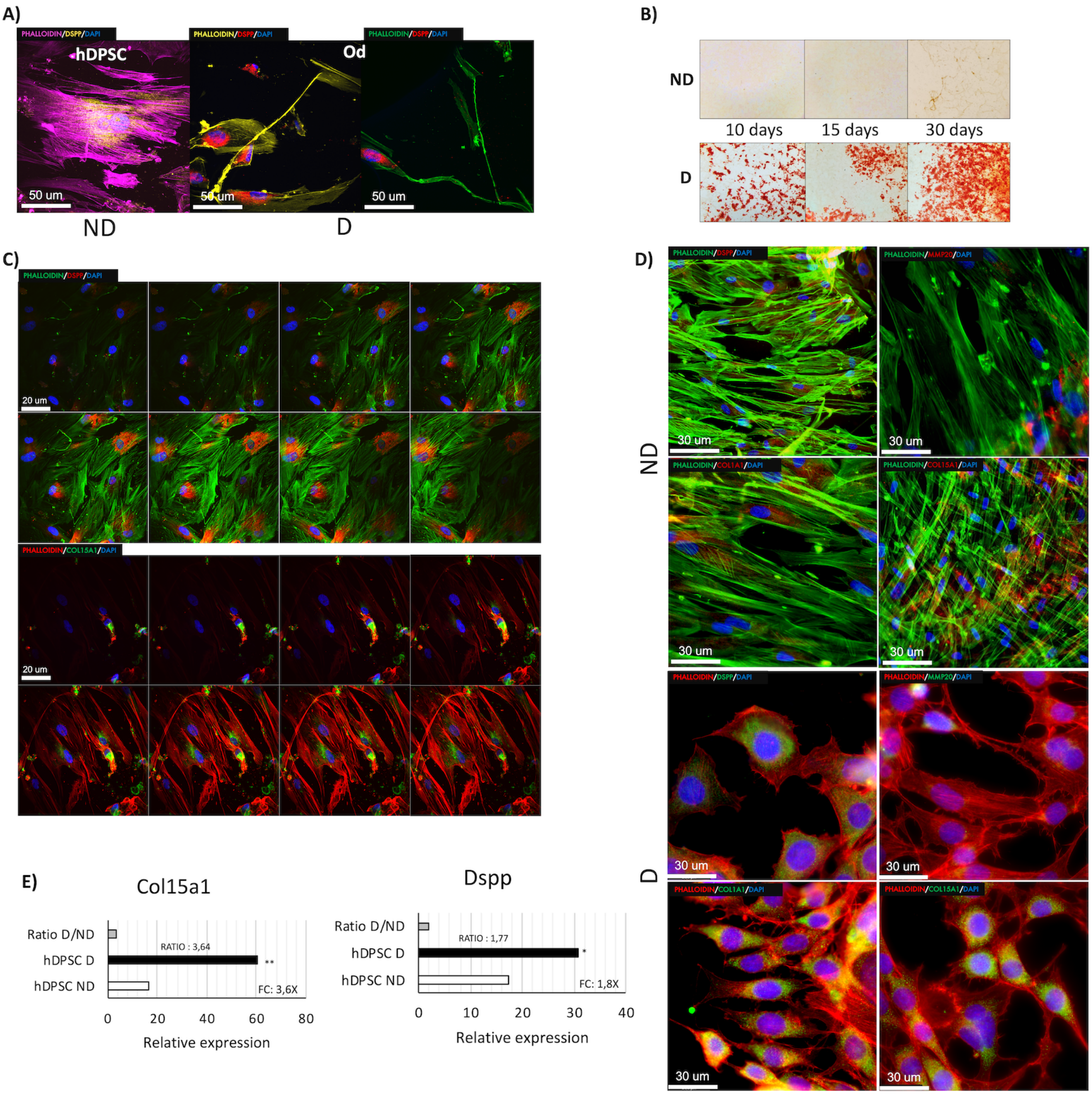

Supplemental figure 5. hDPSC can differentiate into odontoblast-like cells and highly expressed dentinogenesis-related proteins. A. *Immunofluorescent microscopy of non-differentiated (ND) and differentiated (D) hDPSC into odontoblast like-cells. hDPSC are typically ectomesenchymal cells of a spread-out shape before differentiation.: Phalloidin: magenta, yellow and green respectively, DSPP: in yellow and red and DAPI for nucleus: blue. scale bars are represented in each caption).* *Mineralization evaluation by Alizarin Red test for non-differentiated hDPSC (ND) and differentiated hDPSC (D). Two groups of hDPSC were grown separately in normal culture medium and in differentiation medium. Alizarin red tests were performed at different times of culture (10 days, 15 days and 30*

*days). **(C)** DSPP (red) and COLXVA1 (green) protein expression by immunofluorescence of ND and D hDPSC visualized with confocal spinning disk microscopy. Phalloidin for actin (green in upper images and red in lower images) and DAPI for nucleus (blue). **(D)** MMP20, DSPP, COL1A1 and COLXVA1 protein expression by immunofluorescence of ND- and D-hDPSC. Immunofluorescence was performed to study the expression of MMP20, DSPP, COLIA1 and COLXVA1. Phalloidin for actin (green and red) and DAPI for nucleus (blue). The expression of these four main proteins implicated in dentinogenesis was observed on ND-hDPSC (red) and D-hDPSC (green). Magnification for all images was 40x and scale bars are represented on each capture. **(E)** RNA relative expressionby . RTqPCR was performed on RNA extracted from ND- and D-hDPSC after 21 days. Resulted were plotted on a graph with the ratio between D and ND hDPSC. Fold-change for DSPP and COLXVA1 was calculated based on mRNA relative expression of both cell groups. ANOVA test was performed and p-value was < 0.05.*

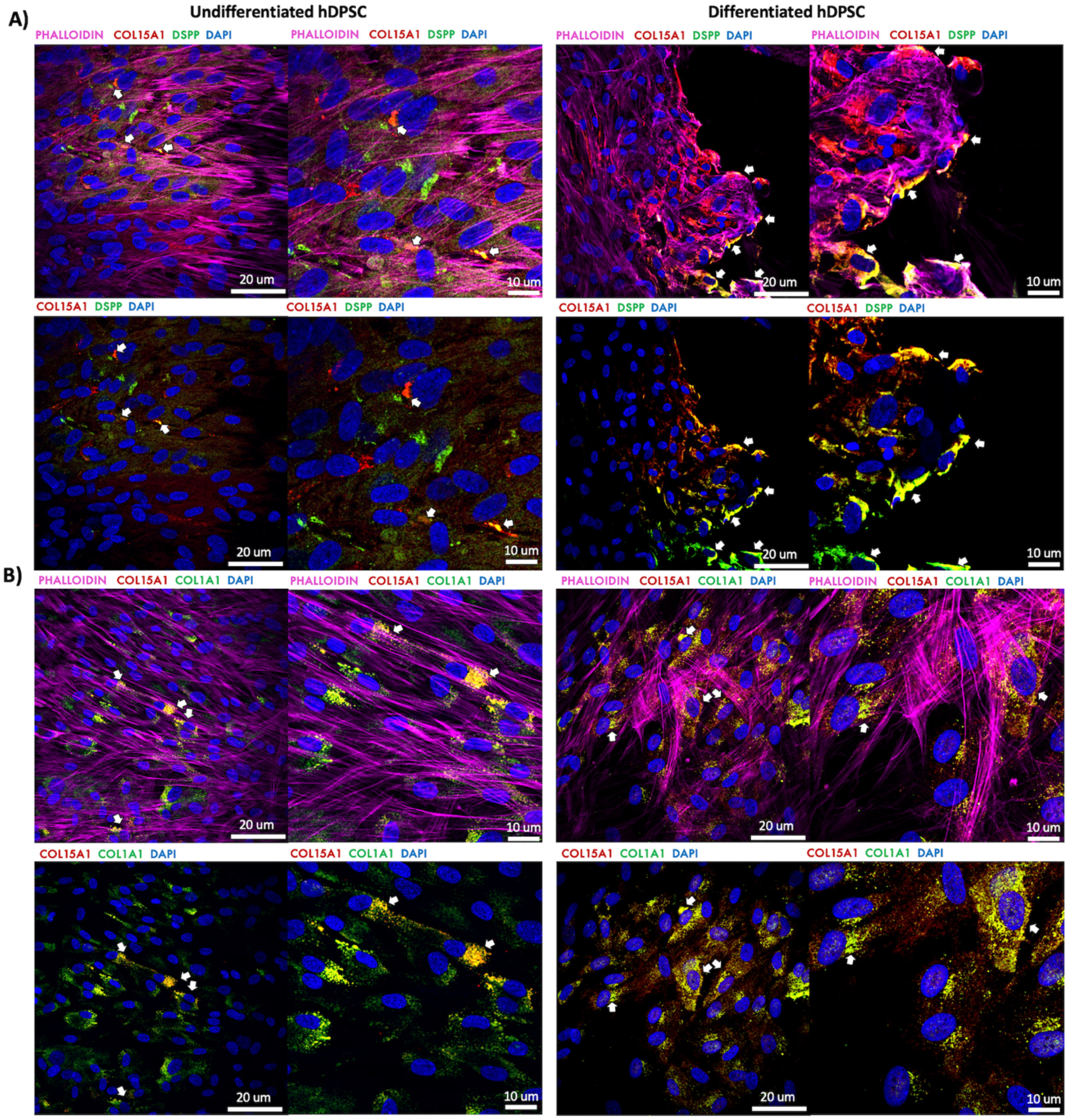

Supplemental figure 6. COLXVA1 co-localise with DSPP and COL1A1 in hDPSC under odontogenic differentiation. *Spinning disk Confocal Immunofluorescent microscopy of non-differentiated (ND) and differentiated (D) hDPSC into odontoblast like-cells.:* ***A.*** *Phalloidin: magenta, DSPP: in green, COLXVA1 in red, and DAPI for nucleus: blue. scale bars represented in each caption. White arrows indicate protein co-localized expression.* ***B.*** *Phalloidin: magenta, COL1A1: in green, COLXVA1 in red, and DAPI for nucleus: blue. scale bars represented in each caption. White arrows indicate protein co-localized expression.*

COLXVA1 loss-of-function blocks mineralization and modulates dentinogenesis-related proteins during odontoblast differentiation from hDPSC

In order to better assess the role of COLXVA1 in dentinogenesis, we created a cellular model for *COLVXA1* loss-of-function by siRNA transduction. *DSPP* RNA interference was performed in parallel as a positive control (**Sup. Fig. 7A**). The impact of *COLXVA1* and *DSPP* inactivation via siRNA on protein expression was evaluated by immunofluorescence in ND- and D-hDPSC (**Sup. Fig. 7B-D**). RNA interference was further validated at the protein level for DSPP (**Sup. Fig. 7C**) and at the mRNA level by RTqPCR for *COLXVA1* **(Sup. Fig. 7D)**. Not surprisingly, DSPP protein expression in D-hDPSC transfected with siDSPP was significantly decreased. Interestingly, DSPP protein was significantly higher in D-hDPSC after siCOLXVA1 transfection (**Sup. Fig. 7C)**. siRNA targeting of either *DSPP* or *COLXVA1* also produced an increase of *COLIA1* mRNA levels in ND- and D-hDPSC and *COLIIIA1* mRNA levels in ND-hDPSC (**Sup. Fig. 7E**). RNA interference against *COLXVA1* and *DSPP* mRNA was temporary, present only 3-to-4 days after transfection (**Sup. Fig. 7F**), allowing cells to recover their normal phenotype. In spite of the recovery of the targeted factors over time, the cells' ability to produce mineralized ECM was negatively impacted by RNA interference of either *DSPP* or *COLXVA1* (**Sup. Fig. 7C-F**), which in some cases also increased ectopic mineralization. Alizarin staining was plotted according to color intensity and showed aberrant production of calcium deposits after 72 hours of transfection and inhibition of *COLXVA1* and *DSPP* mRNA expression **(Sup. Fig. 7F**). Quantitatively, less mineralization spots and coloration were observed with a slower recovery of normal mineralization over time, which more clearly stated a different behavior when compared with *COLXVA1* inhibition **(Sup. Fig. 7A-F**).

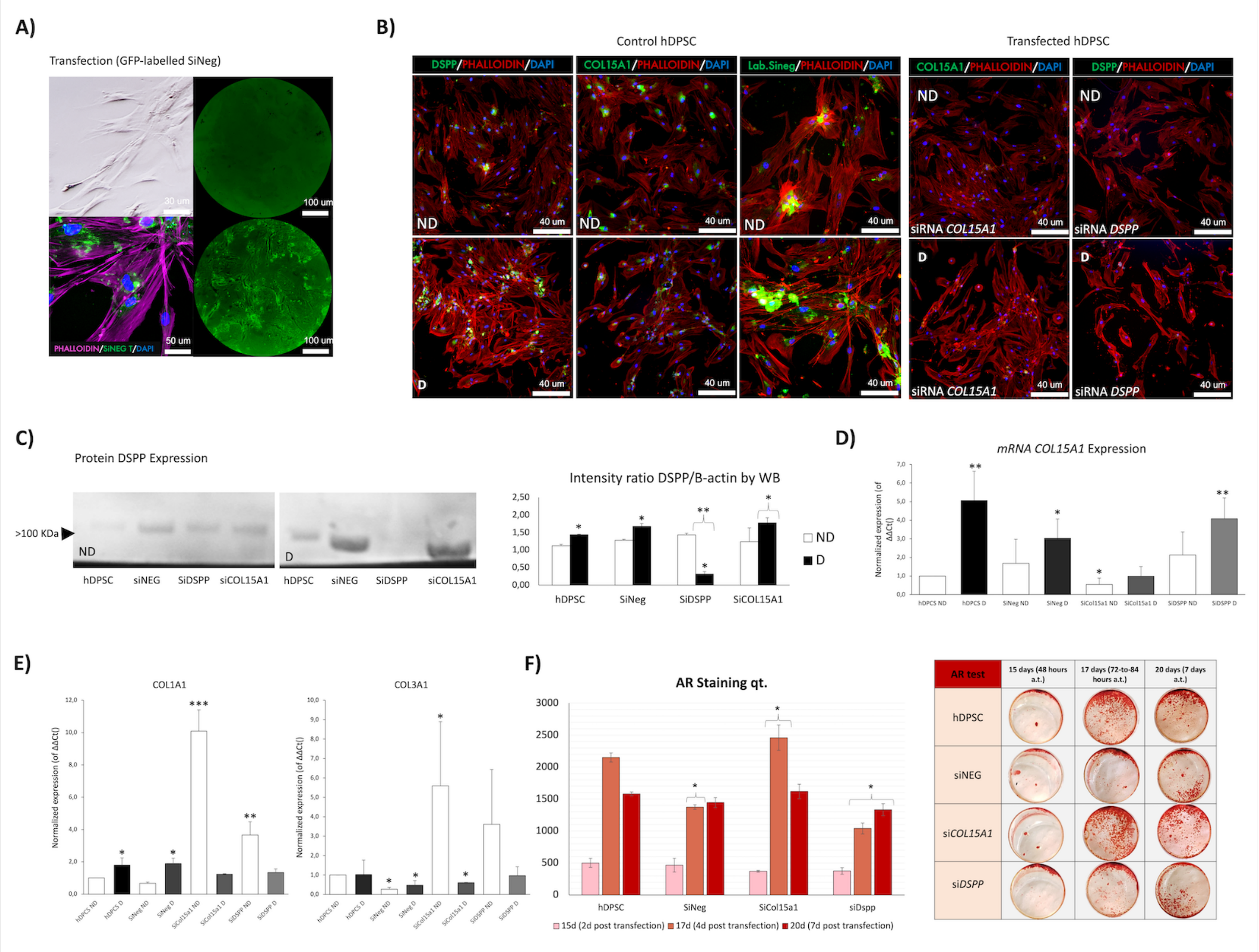

***Supplemental figure 7. Inhibition of ColXVa1 expression affects mineralization and modulates dentinogenesis related proteins in odontoblast-like cells differentiated from hDPSC. A.** Microscopy observations of green labelled negative control of transfection. The transfection of the cells was controlled with Green-labelled siNEG. Non-transfected cells did not show any siNEG signal. hDPCS survived the transfection and the siNEG was observed inside the cell through immuno-fluorescence microscopy. **B.** DSPP and COLXVA1 protein expression by immunofluorescence of ND and D hDPSC transfected with siRNA. Immunofluorescence was performed on DSPP, COLXVA1, siNEG (in green), phalloidin for actin (in red) and DAPI for nucleus (in blue) for ND and D hDPSC before and after transfection. Magnification for all images was 40x and scale bars are represented on each capture. **C.** Western blot on transfected cells for DSPP. Non-differentiated (ND) and differentiated (D) hDPSC were transfected with siNEG, siDSPP and siCOLXVA1. Proteins were extracted from cells 72h after transfection. The intensity ratio of DSPP on B-actin were plotted for ND and D hDPSC without transfection, and transfected with siNEG, siDSPP and siCOL15A1. **D.** COLXVA1 mRNA expression by RTqPCR of ND and D hDPSC transfected with siCOL15A1 and siDSPP. All mRNA expression were normalized with GADPH. **E.** COLIA1 and COLIIIA1 mRNA expression by RTqPCR of ND and D hDPSC transfected with siCOLXVA1 and siDSPP. All mRNA expression were normalized with GADPH. **F.** Mineralization evaluation with Alizarin Red test for transfected cells after siRNA COLXVA1 and DSPP transfection. Alizarin test were performed at 3 (day 14 of differentiation), 6 (day 16 of differentiation) and 8 (day 18 of differentiation) days after transfection of D hDPSC, transfected*

with siNEG, with SiCOLXVA1 and with siDSPP. Staining intensities were represented using pixels intensity values and plotted.

The *COLXVA1* interactome highlights networks in odontogenesis and tooth mineralization.

An interactome of COLXVA1 was performed building a full protein network using ReactomeFIViz app in Cytoscape and interactions were classified following the KEGG Markup Language (KGML) hierarchical structure into four categories: ECrel, PCrel, GErel and PPrel interactions (edges). The full network for genes involved in bone and odontogenesis showed 495 nodes and 2937 interactions for COLXVA1. Direct interactors with COLXVA1 are proteins involved in both bone and tooth morphogenesis. Most proteins interacting with COLXVA1, included cell adhesion and integrin-mediated response molecules as ITGA/B complexes or PTK2 complex, other structural collagens, and also odontogenesis related proteins, including Laminin, Nectin, among others (**Sup. Fig. 8A-C, Sup. Table 3**). After analysis of interactomes (*homo sapiens)*, it appears that COL15A (which is homologous to COL18 and COL4) is linked to neighboring co-expression with MMP20, a gene important in enamel matrix proteins production. Hypothetically COLXVA1 alterations may lead to impaired basement-membrane collagen structure, but also impaired metalloproteinase activity (via MMP20) with implications in both dentinogenesis and amelogenesis during tooth development.

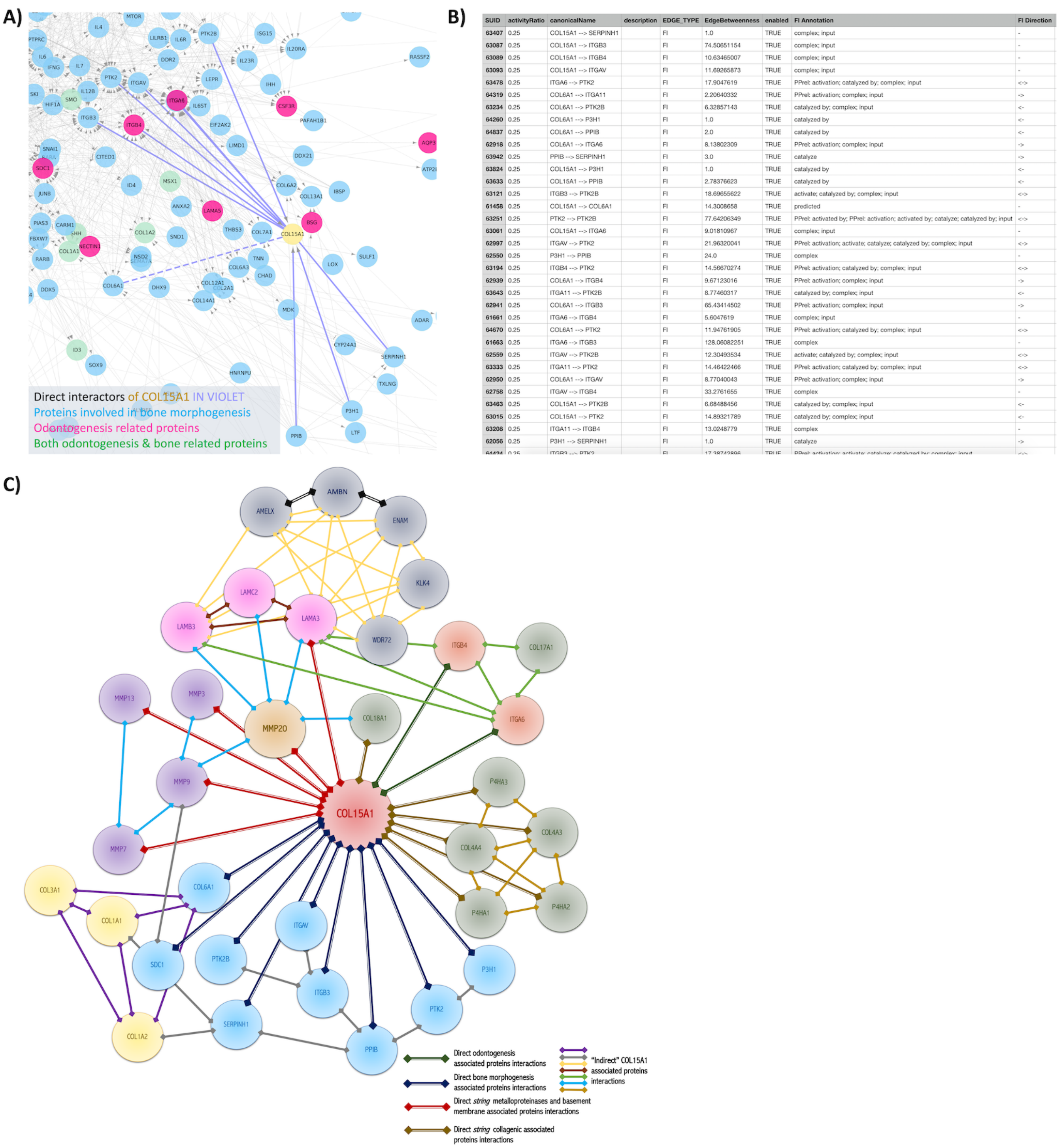

Supplemental Figure 8. Full network of COLXVA1 and genes involved in bone and Odontogenesis: 495 nodes, 2937 interactions.

*(**A, B**) The regulatory network was built using ReactomeFIViz app in Cytoscape. Each interaction also contains a attribute name specifying the type of interaction. COLXVA1 (yellow circle) physically interacts with various factors in violet. Proteins involved in bone morphogenesis are in blue. Odontogenesis related proteins are marked in pink, and both odontogenesis & bone related proteins in green. (**C**) Protein-protein interaction networks functional enrichment analysis constructed through STRING DATABASE. Known and predicted protein-protein interactions by STRING were linked to COL18, COL4*

and MMP20 interactomes (Licensing and credits : Attribution 4.0 International (CC BY 4.0), https://string-db.org/cgi/access?footer_active_subpage=licensing).

An emerging and growing role of COLXVA1 in osteogenesis and dentinogenesis

COLXVA1 is highly expressed in tissues including heart and skeletal smooth muscle. *ColXVa1*$^{-/-}$ deficient mice present both muscle and vascular defects **(36,37).** Studies in mice have also implicated COLXVA1 in muscle function and its targeted inactivation causes skeletal myopathy and cardiovascular defects **(37)**. Neither dental nor bone defects have been reported in *Col1XVa1*$^{-/-}$ deficient mice **(9,10)**. In zebrafish, *ColXVa1* deficiency produces impaired notochord and muscle development **(11)**. Likewise, analogous human *COLXVA1* mutations have not reported mineralized tissues defects **(6–8)**. However, *COLXVA1* also appears to be critical during mineralization of dentin **(5–7,12)** consistent with our expression data. In fact, COLXVA1 is associated with muscular, vascular, neuronal and some epithelial basement membranes, but generally, major producers of COLXVA1 are mesenchymal cells such as fibroblasts, muscle cells, endothelial cells, and osteoblasts as well as odontoblasts **(20, 36, 37, 40).** Bone and dentin are tissues similar in their composition and in their mechanisms of formation. In both processes, the mineralization will start by the differentiation of cells from a mesenchymal origin. Odontoblasts will differentiate from dental pulp cells and osteoblasts arise from bone marrow stem cells. After differentiation, both cell types deposit a non-mineralized, type I collagen-rich matrix called predentin in teeth and osteoid in bone **(13)**. The last step is a maturation of this newly synthetized matrix. The role of COLXVA1 in bone or dentin mineralization is not known yet.

The COLXVA1 mutation identified in the affected family is implicated in this specific phenotype

In this study we present a collection of data implicating a *COLXVA1* mutation in patient phenotypes. We identified, in the described family, a splicing site of exon 20 of *COLXVA1*, as a dominant variant. The critical roles of 29 distinct collagens that have been identified in vertebrate tissues have been clearly illustrated by the wide spectrum of diseases caused by the more than 1,000 mutations that have ,thus far, been identified in more than 20 genes **(14–16)**. These diseases include osteogenesis imperfecta (COL1) with sometimes associated dentinogenesis imperfecta, many chondrodysplasias, several subtypes of Ehlers-Danlos syndrome (mutations

in COL3), Alport syndrome, Bethlem myopathy, certain subtypes of epidermolysis bullosa, Knobloch syndrome and some cases of osteoporosis, arterial aneurysms, osteoarthrosis, and intervertebral disc disease **(11,12,17,19,44)**. The characterization of mutations in distinct collagen genes will systematically add further diseases to this list. Mice with genetically engineered collagen mutations and cellular functional models have proved valuable for defining the functions of various collagens and for studying many aspects of the related diseases **(20,36–38,40).** Hyperlaxity of joints and the dysmorphic sign on the feet "hallux varus" are clinical signs which have been associated to others collagen mutations, as in Ehlers-Danlos syndrome collagen 1,3,5-associated phenotypes **(14,16–18)**. Our affected individuals also complained about skin injuries when exposed to low temperatures, mainly on fingers and toes, sign known as frostbite and an increasing severity of these lesions over time; this type of injury has been associated to vascular consequent injuries and their incapacity to easily regenerate vascularization on extremities. After examination by the cardiovascular specialist, no vascular anomalies were diagnosed, but the increasing frostbite reported by several members of this family could be explained by the documented role of COLXVA1 on vascular physiology and homeostasis **(36,37,40)**. On the other hand, COLXVA1 has also been reported to be expressed during osteogenesis and bone mineralization and our data have shown the expression of COLXVA1 in odontoblast-like cells during differentiation and mineralization and it was also expressed on these cells when modifying DSPP protein expression or related increase of COLIA1 and COLIIIA1. It shows the presence of this gene and collagen at the right time and place to have a role during dentin development, that could explain the impaired dentinogenesis in affected members of this family.

A model where inhibition of COLXVA1 function induced ectopic mineralization and modulated dentinogenesis

Functional evaluation of *in vitro* inactivation, via siRNA transfection of COLXVA1, shows altered and ectopic mineralization. Our data demonstrated that COLXVA1 in odontoblast-like cells leads to an enhancement of mineralization spots and ectopic deposits of calcium, which correlates with patient phenotypes, characterized by a peculiar mineralization of dentinal tubules and numerous calcified odontoblastic processes with mineralized peri- or inter-tubular dentin but without collagen fibers as shown by SEM (**Sup. Fig. 3**). Based on our data, siRNA

inhibiting *COLXVA1* can induce increase in COL1A1 and COL3A1 amounts, potentially producing bone or dentin mineralization defects; this could be a compensatory mechanism to be further investigated. While these *COLXVA1* gene targets may directly alter predentin and osteoid matrix formation and maturation, we can only speculate on a possible model. An increase on these two collagens could produce deficiency in the maturation and evolution of the collagenous matrix into mineralized hydroxyapatite matrix, but currently no reported gains of function mutations in collagens support this premise **(14,16)**. COLXVA1 expression is high during osteogenesis induction and matrix secretion and is then decreased, once the calcified matrix is formed **(12)**. Hypothetically, COLXVA1 could play a role in regulation of hydroxyapatite matrix deposits within the extracellular matrix (ECM)- as mutations of other collagens affect bone mineralization **(12,16,19,20)**. We transfected odontoblast-like cells to inhibit protein expression of COLXVA1 isoform and DSPP (**Sup. Fig. 6**). Blocking DSPP by siRNA knockdown approach dysregulated dentinogenesis and mineralization, decreasing *COLIA1, COLIIIA1,* and *COLXVA1* expression (**Sup. Fig. 5, 6**). Potentially, COLXVA1 would not participate as a minor structural protein in the predentin matrix, but rather as an organizer establishing a mature dentin matrix and facilitating mineralization *via* hydroxyapatite deposits at the right time and place. Impaired expression of COLIA1 and COLIIIA1 (the main proteins of the non-mineralized matrix) could contribute to dentin defects. In fact, during osteogenic differentiation and consequent mineralization, a down-regulation of *COLIA1* and *RUNX2* (observed by RNA sequencing) was associated with an up-regulation of *COLXVA1* **(5,6,21)**. We showed during odontoblasts differentiation, that COLXVA1 siRNA knockdown slightly increased and produced ectopic overall mineralization. Combined with patient phenotypic data COLXVA1 clearly regulates the formation of the mineralized dentin.

Basement membrane components may play an important role on cell behavior and pre-mineralized matrix, as predentin.

The basement membrane (BM) is a specialized ECM structure which delimits tissues compartments, provide structural support and influence cell behavior and cell-to-cell communication **(14,22)**. Basement membrane components are key players in specialized extracellular matrices and have been previously considered as passive scaffolds segregating polarized cells, such as epithelial or endothelial cells, from the underlying mesenchyme, but they play

a multitude of roles from blood filtration to different biomechanical and biochemical properties (14,23–26). Mutations affecting basement membrane components, as Laminin, Collagen IV or ODAM, can directly affect odontogenesis (14,25–27). Numerous mutations in BM collagens genes are associated with a wide variety of genetic diseases **(14,22)**. In fact, COL15 and COL18 are multiplexin (multiple triple-helix domains with interruptions) collagens that are structurally closely related heparan sulphate proteoglycan and both structurally similar molecules with anti-angiogenic fragments. Although, recent data have shown that for COL15 this is mediated via its restin fragment **(28,29)**. COL18 multiplexin protein is embedded into the BM by its C-terminal domain that binds perlecan and laminin compounds, while its N-terminal domain extends into the ECM. Specific COL15 spatial structure and functional interactions have not been elucidated **(14)**. Despite these structural similarities, these collagens have different functions. Dominant missense mutations occur across all collagen types and results in protein misfolding. Some of the physiopathological mechanisms of collagen dominant mutations is the ER retention and ER stress or autophagy induction, which represent potential convergent disease mechanisms for several collagens malfunction and BM defects. For example, COL4 dominant mutations induce activation of autophagy in patient fibroblasts, or COL6 mutations lead to failure to induce autophagy (via an as yet unknown mechanism) and result in mitochondrial defects and production of reactive oxygen species (ROS). Matrix defects, resulting from *COL15* deficiency also cause mitochondrial defects and ROS production **(14,23)**. In fact, *COLXVA1* mutations have been associated to a wide range of diseases **(Sup. Fig. 8) (9,14,22,28,29).**

COLXVA1 interactions show other interactions potentially regulating odontogenesis

Interestingly, several molecules of cell adhesion and integrin-mediated response, such as ITGA and ITGB complexes or PTK2 proteins and even BM collagens such as COLVI are described to be directly associated with the functions and expression of COLXVA1 (**Sup. Fig. 8**). *ColXVa1* gene is highly expressed in muscular and support tissues (in the BM), and in the extracellular matrices of bone and dentin **(9,14,22) (Fig. 4, 5, 6, 7)**. This explains why non-dental anomalies also occur. The splicing mutation of *COLXVA1* may alter signaling pathways related to hard tissues and fibrous connective tissue formation first via the BM, the later by altering mineralization (**Sup. Fig. 8, 9**). More deleterious effects on cardiovascular and muscular

tissues are not associated with this type of *COLXVA1* mutation, but with other clinical variants explaining specific phenotypes **(26,36–38,40,54)**.

Finally, COLXVA1 occurs widely in the basement membrane zones of various tissues. Following recent work and our bioinformatic and functional analysis, the role of this COLXVA1 variant shed light on the wide spectrum of associated phenotypes helping us to better understand its implication on several tissues and their biological functions, from muscular development and dynamics, vascular degeneration to mineralization of bone and tooth. Eklund et al., has induced a COLXVA1 full knock-out mouse line to understand the biological role of this protein **(10)**. Notwithstanding the full lack of COLXVA1, the mutant mice developed and reproduced normally, but they showed progressive histological changes characteristic for muscular diseases after 3 months of age, and they were more vulnerable to exercise-induced muscle injury. In addition, and coming into resonance with individuals described in this article, the full lack of COLXVA1 in mice, did not initially show an abnormal development of the vasculature; moreover, collapsed capillaries and endothelial cell degeneration in heart and skeletal muscles were only observed with age **(9,10)**. Furthermore, this recently described full knock-out mouse model showed that COLXVA1 appears to function as a structural component needed to stabilize skeletal muscle cells and microvessels. Affected individuals of this reported family, in addition to the dental phenotype, with ectopic and abnormal dentin mineralization, altered development of the dental and root anatomy, have described an evolving frostbite on fingers and toes with age. Microvascular ″injuries ″on extremities will need to be further characterized as previous cardiovascular specialist examination of all affected individuals did not unravel vascular defects **(9,10,16,26).** This again supports our postulate of the increasing roles and importance of identified mutations in this collagen 15 (here a splicing site at the terminus of exon 19 and upstream of the collagen domain of this gene inducing exon 20 skipping) and how broad the associated phenotypic spectra could be.

Detailed Materials and Methods

Patients

Affected members of the family were examined at the Reference Centers for Rare oro-dental diseases (O-Rares network) of Strasbourg and Nancy (France). The oral phenotype was documented using the D[4]/phenodent registry protocol **(31)**. This clinical study is registered at https://clinicaltrials.gov: NCT01746121/NCT02397824 and with the French Ministry of Higher Education and Research Bioethics Commission as a biological collection "Orodental Manifestations of Rare Diseases" DC-2012-1677/DC-2012-1002; it was acknowledged by the person protection committee. The parents gave written informed consents for the genetic analyses performed on the salivary samples (OG-250 Oragene®DNA kit, DNA Genotek Inc., Ottawa, Ont., Canada, www.dnagenotek.com) both for them and their children in accordance with the Declaration of Helsinki. They also gave written consent participating to D[4]/phenodent registry and to the publication of this article and the clinical images which are presented **(Figure 1A, 1B).** A small piece of gingiva was also taken during a programmed surgery procedure from patient II.2 (**Figure 1**) in order to extract RNA and confirm exon 20 skipping.

Whole-Exome Sequencing

Whole-exome sequencing (WES) was performed on the affected individuals (II.1, III.1 and III.2) by Integragen (Evry, France, 2014). Exons were captured using SureSelect Human All Exon Kits (Agilent, France) with the company's probe library (Agilent Human All Exon v5 + UTR 75 Mb Kit) and sequenced with an Illumina HISEQ2000 (Illumina, USA) as paired-end 75 bp reads, resulting in an average coverage of 80X.

Gene	gNomen	cNomen	pNomen
COL15A1	Chr9(GRCh37):g.101798451G>C	NM_001855.4:c.2290-1G>C	p.?
ECI2/C6orf201	Chr6(GRCh37):g.4126439G>C	NM_001166010.1:c.514C>G	p.Leu172Val
DNAJC10	Chr2(GRCh37):g.183622522C>A	NM_018981.2:c.1913C>A	p.Pro638His
C7orf50	Chr7(GRCh37):g.1049689G>A	NM_001318252.1:c.220C>T	p.Arg74Trp
PLXDC2	Chr10(GRCh37):g.20357145G>A	NM_001282736.1:c.371G>A	p.Arg124His
CCDC174	Chr3(GRCh37):g.14695975T>A	NM_016474.4:c.85T>A	p.Phe29Ile
COL3A1	Chr2(GRCh37):g.189875017A>C	NM_000090.3:c.3937A>C	p.Lys1313Gln
TTN/-AS1	Chr2(GRCh37):g.179425550C>T	NM_001267550.1:c.85309G>A	p.Gly28437Ser
EML3	Chr11(GRCh37):g.62376557C>T	NM_153265.2:c.806G>A	p.Arg269His
FAM135A	Chr6(GRCh37):g.71234640T>C	NM_001162529.1:c.1853T>C	p.Leu618Pro
CD248	Chr11(GRCh37):g.66082338G>A	NM_020404.2:c.2161C>T	p.Arg721Cys
CSAD	Chr12(GRCh37):g.53566174A>T	NM_015989.4:c.381T>A	p.His127Gln
VPS13A	Chr9(GRCh37):g.79931168C>T	NM_033305.2:c.4709C>T	p.Thr1570Ile
LRIG2	Chr1(GRCh37):g.113637017C>T	NM_001312686.1:c.263C>T	p.Ser88Leu
SPTA1	Chr1(GRCh37):g.158612638C>A	NM_003126.2:c.4571G>T	p.Cys1524Phe
MTOR	Chr1(GRCh37):g.11189856C>T	NM_004958.3:c.5653G>A	p.Val1885Ile
CPZ	Chr4(GRCh37):g.8607794A>G	NM_003652.3:c.755A>G	p.Gln252Arg
TDRD3	Chr13(GRCh37):g.61059936T>C	NM_001146071.1:c.292T>C	p.Cys98Arg
TFB2M	Chr1(GRCh37):g.246707851G>A	NM_022366.2:c.991C>T	p.Arg331Cys
PTPRC	Chr1(GRCh37):g.198721796A>C	NM_002838.4:c.3404A>C	p.Lys1135Thr
LDB3	Chr10(GRCh37):g.88441535G>A	NM_001171610.1:c.664G>A	p.Ala222Thr
KLHDC8A	Chr1(GRCh37):g.205307683G>A	NM_001271863.1:c.799C>T	p.Arg267Trp
RBMXL2	Chr11(GRCh37):g.7111255T>C	NM_014469.4:c.904T>C	p.Tyr302His
SERINC4/SERF2	Chr15(GRCh37):g.44089343T>C	NM_001258031.1:c.844A>G	p.Lys282Glu
WBSCR22	Chr7(GRCh37):g.73100975G>A	NM_001202560.2:c.96G>A	p.Met32Ile
MLLT10	Chr10(GRCh37):g.21823619C>T	NM_004641.3:c.46C>T	p.His16Tyr
FLNC	Chr7(GRCh37):g.128485216C>A	NM_001458.4:c.3697C>A	p.His1233Asn
KCTD8	Chr4(GRCh37):g.44177114T>C	NM_198353.2:c.1115A>G	p.Asn372Ser
TMEM245	Chr9(GRCh37):g.111819564C>A	NM_032012.3:c.1761G>T	p.Leu587Phe
CLK2	Chr1(GRCh37):g.155240714G>T	NM_001294338.1:c.55C>A	p.Arg19Ser
TM9SF4	Chr20(GRCh37):g.30730905G>C	NM_014742.3:c.649G>C	p.Glu217Gln
TRIM66	Chr11(GRCh37):g.8642663T>C	NM_014818.1:c.2933A>G	p.Asn978Ser
CCDC168	Chr13(GRCh37):g.103385998C>G	NM_001146197.1:c.17049G>C	p.Lys5683Asn
CCDC88C	Chr14(GRCh37):g.91780445G>A	NM_001080414.3:c.1715C>T	p.Ser572Leu
POLE2	Chr14(GRCh37):g.50117111A>G	NM_002692.3:c.1369T>C	p.Tyr457His
ROBO4	Chr11(GRCh37):g.124757104G>A	NM_019055.5:c.2204C>T	p.Pro735Leu
FGF4	Chr11(GRCh37):g.69588175G>A	NM_002007.2:c.523C>T	p.Pro175Ser
MRVI1/-AS1	Chr11(GRCh37):g.10597900A>T	NM_130385.3:c.2718T>A	p.His906Gln
AKAP13	Chr15(GRCh37):g.86225419A>G	NM_006738.5:c.5144A>G	p.Asn1715Ser
ANKEF1/SNAP25-AS1	Chr20(GRCh37):g.10030280A>G	NM_022096.5:c.1063A>G	p.Ser355Gly
CBS/L	Chr21(GRCh37):g.44474003C>T	NM_001321072.1:c.1328G>A	p.Arg443Gln
C1QTNF6	Chr22(GRCh37):g.37581353G>C	NM_031910.3:c.194C>G	p.Pro65Arg
CCDC70	Chr13(GRCh37):g.52439779G>A	NM_031290.2:c.265G>A	p.Glu89Lys
SLCO6A1	Chr5(GRCh37):g.101816007A>G	NM_173488.4:c.490T>C	p.Phe164Leu
C3orf20	Chr3(GRCh37):g.14768516A>G	NM_032137.4:c.1675A>G	p.Ile559Val
CDK10	Chr16(GRCh37):g.89753128C>G	NM_052988.4:c.10C>G	p.Pro4Ala
F13A1	Chr6(GRCh37):g.6175022A>T	NM_000129.3:c.1538T>A	p.Met513Lys
MSLNL	Chr16(GRCh37):g.830094T>C	NM_001025190.1:c.907A>G	p.Lys303Glu
ARHGEF12	Chr11(GRCh37):g.120352130A>G	NM_015313.2:c.4399A>G	p.Ile1467Val
SYT8	Chr11(GRCh37):g.1857428G>A	NM_001290332.1:c.475G>A	p.Val159Ile
SPATC1	Chr8(GRCh37):g.145095307G>A	NM_198572.2:c.709G>A	p.Gly237Arg
HERPUD1	Chr16(GRCh37):g.56969215G>T	NM_014685.3:c.216G>T	p.Leu72Phe
ERRFI1	Chr1(GRCh37):g.8073686A>C	NM_018948.3:c.973T>G	p.Leu325Val

Supplemental Table 1. List of remaining heterozygous variants after filtration.

Bioinformatics tools and exome analysis

Burrows-Wheeler Aligner (BWA V7.12) **(32)** was used to align reads on the GRCh37 human genome reference. SNV (single nucleotide variants) and indel (insertion or deletion of bases) were identified and selected using the HaplotypeCaller module of the Genome Analysis ToolKit (GATK, v.3.4.46) (DePristo et al., 2011) and structural variant by CANOES (Backenroth et al., 2014). All the indel and SNV were annotated and ranked using VaRank (Geoffroy et al., 2015) and the Alamut Batch software (Interactive Biosoftware, France). Very stringent criteria were applied to filter out non-pathogenic variants. These included (1) excluding variants represented with an allele frequency of more than 1% in public variation databases-including the 1000 Genomes *(33)*, the gnomAD database *(30)*, or our internal exome database, (2) excluding variants in 5′ or 3′ UTR, (3) excluding variants with intronic locations and no prediction of local splice effect, and (4) excluding synonymous variants without pathogenic prediction of a local

splice effect. Annotation of structural variants (SV) were performed by AnnotSV **(34)**. Variant effects on the nearest splice site were predicted using MaxEntScan *(35)*, NNSplice **(36)** and Splice Site Finder *(37)* programs. The remaining variants were manually curated (**Supplemental Table 1**).

Sanger analysis

Primers to amplify the region on interest were designed using Primer 3 (http://frodo.wi.mit.edu/primer3) tool. Primers sequence are reported in **Supplemental Table2** (*COLXVA1*-ex20-for GAGGCTAGATGGAGGTGAGC; *COLXVA1*-ex20-rev GTC(1)CCACAATACTGGCTCCA). The PCR product was then purified, and the bidirectional Sanger sequencing was performed by GATC Sequencing Facilities (Konstanz, Germany). cDNAs migrated in agarose gel for alternative splicing analysis.

Gene	Forward primer	Reverse primer
COL1A1	5'-TGGGAGTGCAAGGATACTCTATATCG-3'	5'-CCCATCCCATCTTCGACGTAC-3'
COL15A1	5'-GGTGACACTGGTTTACCTGGCT-3'	5'-GCCTTTCCAGAGGAATGTCCTC-3'
COL3A	5'-CTGGACCAAAAGGTGATGCT-3'	5'-CAGGGTTTCCATCTCTTCCA-3'
DSPP	5'-CCTAAAGAAAATGAAGATAATT-3'	5'-TAGAAAAACTCTTCCCTCCTAC-3'
GADPH	5'-CCACCCATGGCAAATTCCATGGCA-3'	5'-TCTAGACGGCAGGTCAGGTCCACC-3'
MMP20	5'-TCCATCCCTGACCTCTGTGACT-3'	5'-AGTGAACCTGCCGTCTCCAGAA-3'

Target	Characteristics	Reference
**Ameloblastin**	mouse monoclonal anti-AMBN	sc-271012, Santa Cruz
**Ameloblastin**	rabbit monoclonal anti-AMBN	sc-50534, Santa Cruz
**Amelogenin**	mouse monoclonal anti-AMELX	sc-365284, Santa Cruz
**Enamelin**	goat polyclonal anti-ENAM	sc-33107, Santa Cruz
**COL1A1**	mouse monoclonal anti-COL1A1	sc-293182, Santa Cruz
**MMP20**	rabbit polyclonal anti-MMP20	bs-5788R, Bioss
**FAM83H**	rabbit polyclonal anti-FAM83H	sc-PA5-55094, Thermofisher
**Goat IgG**	Donkey Fluor™ 488	A-11055, Thermofisher
**Rabbit IgG**	Donkey Fluor™ 488	A-21206, Thermofisher
**Mouse IgG**	Donkey Fluor™ 488	A-21202, Thermofisher
**Goat IgG**	Donkey Fluor™ 594	A-11058, Thermofisher
**Rabbit IgG**	Donkey Fluor™ 546	A10040, Thermofisher
**Mouse IgG**	Donkey Fluor™ 568	A10037, Thermofisher

Supplemental Table 2. Primers and Primary antibodies used

Scanning electron microscopy

The specimens (71, 55 and control "primary incisor 62", physiologically shed primary teeth) were stored in 70% ethanol at 4°C until analysis. The samples were sectioned along the sagittal plane using a diamond embedded saw mounted on a microtome (Walter EBNER, Le Locle, Switzerland). Subsequently, 1200, 2400, and 4000 SiC abrasive papers were used, under continuous water irrigation, to polish the surface of the specimens [1]. Then, the polished sections were etched using 20% citric acid for 2 min, rinsed for 10 s with distilled water, and dehydrated in a graded series of ethanol solutions. They were then mounted on aluminum SEM stubs, and sputter-coated with a gold–palladium alloy (20/80 weight %) using a Hummer JR sputtering device (Technics, CA, USA). The morphological and chemical characteristics were determined using a Quanta 250 FEG scanning electron microscope (FEI Company, Eindhoven, The Netherlands) at 10 kV acceleration voltage of the electrons. EDX analysis (n=5) was performed with a working distance of 10 mm and an acquisition time of 60 s **(38)**. The weight percentages of chemical elements of the surfaces of the different dental tissues were obtained.

Tissues preparation

Mice were euthanized with an intra-peritoneal lethal injection of pentobarbital (100 mg/kg). Intra-cardiac perfusion with a fixative solution containing 4% paraformaldehyde in phosphate buffer saline (PBS) pH 7.4 was then performed. Heads and maxillae were then dissected and post-fixed by immersion in the same fixative solution overnight at 4◦C. After rinsing in PBS for 24 hours, heads and maxillae were processed for histologic analysis by decalcification at 4◦C for 3-to-6 weeks in a pH 6.7 solution containing 4.13% EDTA changed twice a week with constant stirring. After extensive washing in PBS, the samples were dehydrated in increasing concentrations of ethanol and toluene and finally embedded in OCT™ (France Gentaur SARL, Paris, France) for frozen sections. Serial frontal sections of the mouse head were cut with a Cryostat™.

In situ hybridization

A full length Col15a1 probe (Dharmacon) was used to generate an antisense probe. In situ hybridization (ISH) was performed using digoxigenin-labeled RNA probes on 12µm frozen sections. The samples were fixed in 4% PFA for 10 min at 4oC, rinsed with PBS, and then dehydrated in graded ethanol (70%, 95%, 100%, and 95%) 5 min each, and dried for 1hr. The probe was diluted in hybridization buffer at a concentration of 1 µg/ml. The probe mix was denatured by a 10-min incubation at 70°C and placed on ice. An aliquot of 100 µl was applied on each slide. The slides with the probe and the coverslip were put in a humidified chamber at 65oC over-night. 5x SSC pre-warmed (70oC) was used to allow coverslips to detach. The slides were then washed 2 times for 30 min at 65°C in 1× standard saline citrate (SSC), 50% formamide, 0.1% Tween-20, and 2 times for 30 min at RT in MABT buffer (1× MAB (Maleic acid buffer): 0.5 m maleic acid (Roche), 0.75 m NaCl, NaOH to ph 7.5 plus 0.1% Tween-20).

Slides were incubated for 1 h at RT with a blocking solution (20% goat serum, 2% blocking reagent in MABT). The anti-DIG antibody was diluted 1:2,500 in blocking solution, and 200 µl was added to each slide, which were covered by Parafilm and incubated overnight at 4°C. Slides were washed 5 times in MABT for 20 min and then 2 times for 10 min in NTMT buffer (100 mm NaCl, 100 mm Tris-HCl pH 9.5, 50 mm MgCl2 · 6 H2O, 0.1% Tween-20). Hybridized probes were visualized with NTMT, BCIP and NBT in PBT.

Cell culture

Human Dental Pulp Stem Cells (hDPSC) are immortalized multipotent stem cells harvested from soft living pulp tissue inside adult teeth. hDPSC were provided (courtesy of Dr. Papagerakis Petros, from The University of Saskatchewan, Canada and Dr. Sylvie Babajko, from Paris, France). They were cultured in DMEM medium + GlutaMAX™-I with 1g/L of D-Glucose and sodium pyruvate (Gibco™; Thermo Fisher Scientific, Illkirch-Graffenstaden, France) supplemented with 15% Fetal Bovine Serum and 1% penicillin-streptomycin at 37°C in a humidified atmosphere with 5% CO2. Culture medium was changed twice a week. The cells were differentiated into odontoblast in a differentiation medium up to 3 weeks. The differentiation medium was composed of previous medium but supplemented with 0.1 µM dexamethasone (Sigma-Aldrich, Darmstadt, Germany), 5 mM β-glycerophosphate (Santa Cruz, Heidelberg, Germany), 50 µg/mL ascorbic acid (Sigma-Aldrich), and 10 ng/mL TGF-β1 (Sigma-Aldrich) at 37°C in a 5% CO2 incubator. Trypsin-EDTA 0,25% was used to harvest the cells.

Immunofluorescence

Immunofluorescence was performed on cells fixed with 4% PFA for 15min at RT. After fixation the cells were washed twice with PBS and then treated with PBS tween 0.2% for 15min, then they were saturated with BSA 0.5% with triton X-100 0.1% in PBD for 15min at RT Primary antibodies are diluted 1/250 in this same buffer and add on the cells for 1h at RT (**Supplemental Table 2).** Two washes were performed with PBS before the 1h incubation at RT of the secondary antibody diluted 1/500 in PBS. The cells were washed twice with PBS for 10min while agitating. The phalloidin solution was used to incubate for 15min, was prepared by adding 30µL of DMSO into the powder from vial, and diluted 1/1000 with PBS (1% BSA). Two more washes with PBS for 5 min with agitation were performed. One drop of DAPI per slide was added and incubated for 5min at RT. One drop of mounting medium was added surmounted by a slat.

Mineralization test with Alizarin Red

Calcium deposits were specifically stained bright orange-red using Alizarin Red solution (REF : A5533, Merck, France). The staining solution was prepared dissolving 2g Alizarin Red

in 100mL distilled water. The pH was adjusted to 4.1-4.3 with 0.1% NH_4OH. The medium of the 24-well plate was discarded and cells washed with PBS. Paraformaldehyde (PFA 4%) was added to cover the cells for 30min. The PFA was removed and the cells were washed with distilled water. The staining solution was added, 200µL per well, and the plate was incubated at RT in the dark for 45min. The Alizarin Red was carefully aspirated and the cell monolayers washed 3 times with distilled water before adding PBS.

High-speed multispectral spinning-disk confocal microscope system for immunofluorescence of cells

We used a multispectral spinning-disk confocal microscope on Inverted Leica DMI8 for bright-field and fluorescence microscopy. The camera employed "Evolve 512: 512X512 pixels"; pixels size :16µm and Orca Flash 4.0: 2048X2018 pixels (pixel size: 6.5µm) and uses a 63x 1,4- HCX PL APO Lambda blue or 100x HC PL APO 1.47-NA objectives lens of oil immersion for high magnification, high resolution, and thus maximal speckle contrast. To construct the figure, contrast and brightness were adjusted and unsharp masking filters were applied to the original gray-scale images from each fluorescence channel. The 12-to-14-bit images were then copied and color combined into 24-to-32-bit RBG images using Metamorph software.

RNA isolation and reverse transcription (mice and cells samples)

Total RNA from cells were extracted using TRIzol™ Reagent (Thermo Fisher Scientific, Illkirch-Graffenstaden, France) until the separation phases steps and then the Rneasy® Plus Micro kit (Qiagen) was used. Briefly, after the medium removal from the 24-well culture plate 400µL of TRIzol™ was added and cells were scratch with a tip. The lysate was taken out and 100µL of PBS was used to rescue the remaining cells of the plate and then mixed. The lysate was incubated 3min with 0.1mL of chloroform and then centrifuged for 15min at 12,000g at 4°C. The aqueous phase containing RNA was taken out from the 2 other phases and place in a gDNA Eliminator spin column. Next steps were performed as the manufacturer recommendation (Rneasy Plus Kits, Cat. No./ID:74034, Qiagen, France). The column was centrifuged for 30s at more than 8,000g. One volume of 70% ethanol was added to the flow-through, mixed by pipetting, transferred to the Rneasy spin column placed in a 2mL collection tube and centrifuged for 15s at more than 8,000g. The flow-through was discarded and 700µL of buffer BW1 was added to the column. This centrifugation step was repeated with first 500µL of buffer RPE followed with 500µL of 80% ethanol. The column was placed in a new collection tube and centrifuged at full speed for 5min. The column was placed in a new collection tube and 14µL of Rnase-free water was added directly to the center of the spin membrane. A last centrifugation for 1min at full speed to elute RNA.

The total RNA concentration was quantified using NanoDrop 1000 (Thermo Fisher Scientific, Illkirch-Graffenstaden, France). The reverse transcription of the mRNA was performed using

the SuperScript™ II RT (Thermo Fisher Scientific, Illkirch-Graffenstaden, France) following the manufacturer instructions. Two mixes were prepared: the first with 250ng of random primer (1µL), 1µg of RNA diluted in 9µL of sterile distilled water and 2µL dNTP (5mM) 2µL; the second was composed of 5X first-strand buffer (4µL), 0.1 M DTT (2µL), RNaseOUT (1µL) and SuperScript (1µL). We heated the mix1 to 65°C for 5min, cool it on ice before adding the mix2 and incubating at 42°C for 50min followed by a 15min step at 70°C. At the end, 40µL of Dnase free water was added.

Quantitative real-time PCR analysis

To quantify mRNA expression, qPCR was performed on cDNA samples. PCR amplification and analysis were performed with the RealPlex 2 qPCR Real Time PCR ThermoCycler (Eppendorf, Illkirch-Graffenstaden, France). Amplification reactions were performed using LightCycler® 480 SYBR Green I Master (Roche Life Science). GAPDH was used as endogenous RNA control (housekeeping gene) in all samples. Primer sequences related to *COLIA1*, *COLXVA1*, *COLIIIA1*, *DSPP*, *GADPH*, and *MMP20* were purchased from Merk, (Great-Britain) (**Table 2**). Expression level was calculated after normalization to the housekeeping *GADPH* gene expression.

siRNA transfection

Transfection was performed following the manufacturer instructions. Briefly, 1µL of Lipofectamine™ 3000 Reagent (Thermo Fisher Scientific, Illkirch-Graffenstaden, France) diluted in 50µL of medium was mixed (ratio 1:1) with 0.5µg of siRNA (Thermo Fisher Scientific, Illkirch-Graffenstaden, France) diluted in 48µL of medium and 2µL of P3000™ Reagent. The mix incubated for 15min at RT before added to the confluent cell in a 24-well plate. This technique was performed simultaneously with siNeg (Ambion Silencer® FAM™ Labeled Negative Control, ThermoFisher) as control for transfection, si*DSPP* (Ambion, *DSPP* Silencer® Select Pre-designed siRNA, ThermoFisher) as positive control and for si *COLXVA1* (Ambion, *COLXVA1* Silencer® Select Pre-designed siRNA, ThermoFisher).

Western blotting

Briefly, cells were lysed for 5 min on ice in 200 µl of ice-cold RIPA buffer (65 mM Tris–HCl, pH 7.4, 150 mM NaCl, and 0.5% sodium deoxycholate) supplemented with phosphatase inhibitor cocktails I and II and a protease inhibitor cocktail (Sigma, Darmstadt, Germany). Lysates were centrifuged at 10,000 g at 4 °C for 10 min, supernatants were collected for quantification using the Bradford protein assay (Bio-Rad, Hercules, CA, USA) and 20 µg of cell lysates were loaded on a 12% SDS-PAGE for each condition. Rabbit anti-human DSPP (bs-10316R, Bioss), and against β-actin (1/2000, mouse IgG) from Santa Cruz Biotechnology (Heidelberg, Germany)

were used for immunolabelling. Secondary alkaline phosphatase conjugated antibodies (anti-mouse (1/3000) or anti-rabbit (1/5000)) were purchased from Bethyl Laboratories (Montgomery, Texas, USA) (**Table 2**).

Full network of COLXVA1 and genes involved in bone and odontogenesis

The regulatory network was built using ReactomeFIViz app in Cytoscape™. The interactions reported by the application (Cytoscape™) are classified hierarchically following the KEGG Markup Language (KGML) structure into four categories: Ecrel, Pcrel, Gerel and Pprel interactions (edges). Each interaction also contains a name attribute specifying the type of interaction. Following PathPPI classification, we should consider only Gerel edges, indicating the source node to be acting like a transcription factor and the target node as a repressed or expressed gene and Pprel edges (protein protein activation or/and inhibition relationships), plus the FI annotated interactions labelled as complexes and predicted to highlight in our networks using different colours for the edges. To further analyse possible interactions of *COLXVA1*, a protein-protein interaction network's functional enrichment analysis was observed on *String Database* (Licensing and credits : Attribution 4.0 International (CC BY 4.0; https://string-db.org/cgi/access?footer_active_subpage=licensing). Finally, all pathways and "go terms" that contained at least 10 of the genes identified by the previous analysis were included in Database for Annotation, Visualization and Integrated Discovery (DAVID) and they were classified by enrichment score to represent on a table the most important KEGG Pathways. (***Sup. Figure 8, Sup. Table 3***).

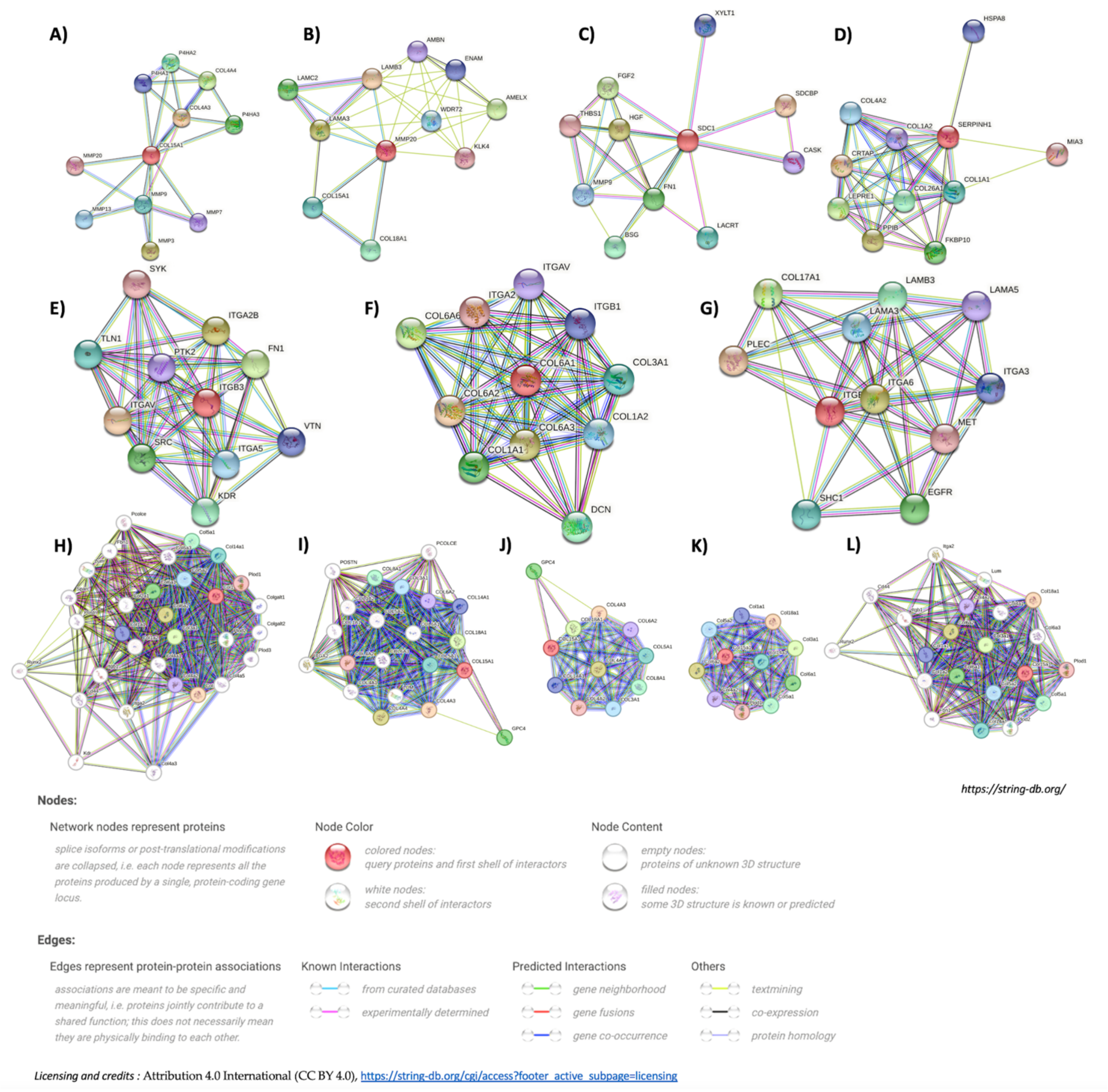

Supplemental Figure 9. *String extracted and analyzed interactomes*

Category	Term	Count	%	Genes	Fold Enrichment	PValue	Bonferroni	Benjamini	FDR
UP_SEQ_FEATURE	REGION:Triple-helical region	11	14.8648649	COL17A1, COL1A1, COL3A1, COL4A2, COL5A1, COL11A1, COL4A4, COL6A2, COL4A3, COL6A1, COL6A3	132.86604	1.84E-19	1.35E-16	6.73E-17	6.09E-17
GOTERM_BP_DIRECT	GO:0035987~endodermal cell differentiation	13	17.5675676	LAMB3, COL11A1, LAMA3, FN1, LAMB1, MMP8, VTN, COL4A2, COL6A1, COL8A1, GRB2, ITGAV, ITGA5	110.23932	4.00E-22	3.03E-19	6.06E-20	5.50E-20
GOTERM_MF_DIRECT	GO:0030020~extracellular matrix structural constituent conferring tensile strength	17	22.972973	COL17A1, COL18A1, COL15A1, COL14A1, COL11A1, COL1A1, COL3A1, COL1A2, COL4A2, COL5A1, COL4A4, COL6A2, COL4A3, COL5A2, COL6A1, COL6A3, COL8A1	99.51820988	1.43E-28	2.23E-26	7.43E-27	5.62E-27
GOTERM_BP_DIRECT	GO:0033627~cell adhesion mediated by integrin	11	14.8648649	ITGB1, VTN, ITGA3, ITGB4, ITGA2, ITGB3, ITGA2B, ITGAV, ITGA6, ITGA5, FBN1	70.52834542	2.95E-16	2.52E-13	3.20E-14	2.90E-14
UP_KW_CELLULAR_COMPONENT	KW-0084~Basement membrane	12	16.2162162	COL17A1, LAMA5, COL18A1, COL4A2, LAMB3, COL4A4, COL4A3, LAMA3, COL8A1, LAMB1, LAMC2, LAMC1	66.63511831	1.43E-17	2.85E-16	9.51E-17	8.56E-17
INTERPRO	IPR008160:Collagen triple helix repeat	18	24.3243243	COL17A1, COL18A1, COL26A1, COL15A1, COL14A1, COL11A1, COL1A1, COL3A1, COL1A2, COL4A2, COL5A1, COL4A4, COL6A2, COL4A3, COL5A2, COL6A1, COL6A3, COL8A1	58.27134146	8.25E-26	1.34E-23	1.34E-23	1.02E-23
GOTERM_MF_DIRECT	GO:0005518~collagen binding	15	20.2702703	CRTAP, VWF, COL14A1, ITGA3, LUM, ITGA2, FN1, P3H1, PCOLCE, GP6, VTN, COL6A2, COL6A1, SERPINH1, CD44	58.10968137	2.83E-21	4.42E-19	1.10E-19	8.35E-20
GOTERM_BP_DIRECT	GO:0030199~collagen fibril organization	16	21.6216216	FKBP10, COL18A1, COL15A1, CRTAP, COL14A1, LUM, COL11A1, P3H1, COL1A1, COL3A1, COL1A2, COL4A2, COL5A1, COL4A3, COL5A2, SERPINH1	55.3428165	2.09E-22	1.59E-19	3.97E-20	3.60E-20
GOTERM_CC_DIRECT	GO:0005604~basement membrane	19	25.6756757	COL17A1, LAMA5, COL18A1, COL15A1, CD151, DST, ITGB4, LAMA3, FN1, LAMB1, LAMC2, LAMC1, VTN, COL4A2, COL5A1, COL4A4, COL4A3, AMTN, FBN1	55.22142455	8.47E-27	1.77E-24	5.90E-25	4.52E-25
UP_KW_DISEASE	KW-0263~Epidermolysis bullosa	10	13.5135135	COL17A1, CD151, LAMB3, DST, ITGA3, ITGB4, LAMA3, ITGA6, LAMC2, PLEC	54.79166667	1.47E-14	3.66E-13	3.66E-13	3.08E-13
GOTERM_CC_DIRECT	GO:0005581~collagen trimer	18	24.3243243	COL17A1, COL18A1, COL26A1, COL15A1, COL14A1, COL11A1, PCOLCE, MMP8, COL1A1, COL3A1, COL1A2, COL4A2, COL5A1, COL6A2, COL4A3, COL6A1, SERPINH1, COL6A3	53.42811961	4.80E-25	1.00E-22	2.51E-23	1.92E-23
KEGG_PATHWAY	hsa04512:ECM-receptor interaction	28	37.8378378	ITGB1, LAMA5, ITGB4, ITGB3, LAMA3, ITGA2B, LAMC2, LAMC1, VTN, ITGAV, LAMB3, VWF, ITGA3, ITGA2, FN1, LAMB1, GP6, COL1A1, COL1A2, COL4A2, COL4A4, COL6A2, COL4A3, COL6A1, COL6A3, ITGA6, ITGA5, CD44	51.31057876	1.11E-41	1.28E-39	6.40E-40	4.73E-40
GOTERM_MF_DIRECT	GO:0005201~extracellular matrix structural constituent	27	36.4864865	COL17A1, LAMA5, COL18A1, COL15A1, COL14A1, COL11A1, LAMA3, LAMC2, LAMC1, VTN, POSTN, LAMB3, VWF, LUM, FN1, LAMB1, PCOLCE, COL1A1, COL3A1, COL1A2, COL4A2, COL5A1, COL4A4, COL4A3, COL5A2, COL8A1, FBN1	51.16996403	3.49E-38	5.45E-36	5.45E-36	4.12E-36
UP_SEQ_FEATURE	MOTIF:Cell attachment site	15	20.2702703	LAMA5, COL18A1, VWF, COL14A1, LAMA3, FN1, COL1A1, VTN, COL4A4, COL6A2, COL4A3, COL5A2, COL6A1, COL6A3, FBN1	46.82204677	8.27E-20	6.04E-17	6.04E-17	5.46E-17
UP_KW_DOMAIN	KW-0176~Collagen	21	28.3783784	COL17A1, COL18A1, COL26A1, COL15A1, COL14A1, COL11A1, PCOLCE, MMP8, COL1A1, COL3A1, COL1A2, COL4A2, COL5A1, COL4A4, COL6A2, COL4A3, COL5A2, COL6A1, SERPINH1, COL6A3, COL8A1	44.16969697	4.99E-28	8.49E-27	8.49E-27	6.99E-27
GOTERM_MF_DIRECT	GO:0005178~integrin binding	26	35.1351351	ITGB1, LAMA5, CD151, ITGB4, SRC, ITGB3, LAMA3, ITGA2B, LAMC1, VTN, ITGAV, VWF, DST, ITGA3, ITGA2, FN1, LAMB1, PTK2, COL3A1, COL5A1, COL4A3, ITGA6, TLN2, ITGA5, TLN1, FBN1	42.80746528	1.43E-34	2.23E-32	1.12E-32	8.45E-33
GOTERM_BP_DIRECT	GO:0007229~integrin-mediated signaling pathway	18	24.3243243	ITGB1, LAMA5, DST, ITGB4, SRC, ITGA3, ITGB3, ITGA2, LAMA3, ITGA2B, FN1, PTK2, COL3A1, ITGAV, ITGA6, ITGA5, TLN1, BCAR1	42.62892622	2.69E-23	2.04E-20	6.79E-21	6.16E-21
GOTERM_BP_DIRECT	GO:0007160~cell-matrix adhesion	16	21.6216216	ITGB1, COL17A1, ITGB4, ITGA3, ITGB3, ITGA2, PXN, ITGA2B, FN1, VTN, COL3A1, ITGAV, ITGA6, ITGA5, CD44, VCL	38.58765187	6.78E-20	5.14E-17	8.56E-18	7.76E-18
GOTERM_BP_DIRECT	GO:0030198~extracellular matrix organization	26	35.1351351	COL17A1, ITGB1, COL18A1, COL15A1, COL14A1, COL11A1, ITGB3, ITGA2B, MMP20, VTN, ITGAV, POSTN, ITGA3, ITGA2, MMP8, COL1A1, COL3A1, COL1A2, COL4A2, COL5A1, COL4A4, COL4A3, COL5A2, COL8A1, ITGA6, ITGA5	35.97283073	1.66E-32	1.26E-29	6.31E-30	5.72E-30
UP_KW_DISEASE	KW-0986~Amelogenesis imperfecta	10	13.5135135	FAM83H, LAMB3, ENAM, AMBN, AMTN, KLK4, WDR72, ODAPH, AMELX, MMP20	35.06666667	2.42E-12	6.06E-11	3.03E-11	2.54E-11
UP_KW_CELLULAR_COMPONENT	KW-0272~Extracellular matrix	36	48.6486486	COL17A1, LAMA5, COL18A1, COL15A1, COL14A1, COL11A1, LAMA3, LAMC2, LAMC1, MMP20, AMELX, POSTN, COL26A1, CRTAP, LAMB3, VWF, LUM, AMBN, FN1, P3H1, LAMB1, MMP8, COL1A1, COL3A1, COL1A2, COL4A2, COL5A1, ENAM, COL4A4, COL6A2, COL4A3, COL5A2, COL6A1, COL6A3, COL8A1, FBN1	31.63969646	1.60E-44	3.19E-43	3.19E-43	2.87E-43
KEGG_PATHWAY	hsa04510:Focal adhesion	38	51.3513514	ITGB1, LAMA5, ITGB4, SRC, ITGB3, PXN, LAMA3, ITGA2B, LAMC2, LAMC1, EGFR, VTN, FLNA, ITGAV, FYN, LAMB3, VWF, ITGA3, ITGA2, FN1, LAMB1, PTK2, COL1A1, COL1A2, COL4A2, COL4A4, COL6A2, COL4A3, COL6A1, COL6A3, GRB2, ITGA6, TLN2, ITGA5, TLN1, SOS1, BCAR1, VCL	30.5299749	5.73E-50	6.59E-48	6.59E-48	4.87E-48
KEGG_PATHWAY	hsa05222:Small cell lung cancer	17	22.972973	ITGB1, LAMA5, LAMB3, ITGA3, ITGA2, LAMA3, ITGA2B, FN1, LAMB1, LAMC2, LAMC1, PTK2, COL4A2, COL4A4, COL4A3, ITGAV, ITGA6	30.13699754	5.19E-20	5.96E-18	9.94E-19	7.35E-19
KEGG_PATHWAY	hsa04974:Protein digestion and absorption	18	24.3243243	COL17A1, COL18A1, COL26A1, COL15A1, COL14A1, COL11A1, COL1A1, COL3A1, COL1A2, COL4A2, COL5A1, COL4A4, COL6A2, COL4A3, COL5A2, COL6A1, COL6A3, COL8A1	28.50192343	7.64E-21	8.78E-19	1.76E-19	1.30E-19
GOTERM_CC_DIRECT	GO:0031012~extracellular matrix	26	35.1351351	COL17A1, COL18A1, COL15A1, COL14A1, COL11A1, MMP20, POSTN, VWF, LUM, FN1, MMP8, COL1A1, COL3A1, COL1A2, COL4A2, COL5A1, ENAM, COL4A4, COL6A2, COL4A3, COL5A2, COL6A1, AMTN, COL6A3, COL8A1, FBN1	28.00907858	1.19E-29	2.49E-27	1.24E-27	9.51E-28
GOTERM_CC_DIRECT	GO:0005788~endoplasmic reticulum lumen	30	40.5405405	COL17A1, FKBP10, COL18A1, COL15A1, COL14A1, COL11A1, LAMC1, SERPINH1, AMELX, COL26A1, CRTAP, AMBN, FN1, P3H1, LAMB1, COL1A1, COL3A1, COL1A2, COL4A2, COL5A1, ENAM, COL4A4, COL6A2, COL4A3, COL5A2, COL6A1, AMTN, COL6A3, COL8A1, FBN1	26.91448683	3.97E-34	8.29E-32	8.29E-32	6.35E-32
KEGG_PATHWAY	hsa05146:Amoebiasis	15	20.2702703	LAMA5, LAMB3, LAMA3, FN1, LAMB1, LAMC2, LAMC1, PTK2, COL1A1, COL3A1, COL1A2, COL4A2, COL4A4, COL4A3, VCL	23.98446171	4.82E-16	5.11E-14	7.92E-15	5.86E-15
BIOCARTA	h_integrinPathway:Integrin Signaling Pathway	10	13.5135135	ITGB1, SRC, PXN, GRB2, FYN, TLN1, SOS1, PTK2, VCL, BCAR1	20.88803089	8.34E-11	6.84E-09	6.84E-09	4.92E-09
UP_KW_PTM	KW-0379~Hydroxylation	21	28.3783784	COL17A1, COL18A1, COL26A1, COL15A1, CRTAP, COL14A1, AMBN, COL11A1, EGFR, COL1A1, COL3A1, COL1A2, COL4A2, COL5A1, COL4A4, COL6A2, COL4A3, COL5A2, COL6A1, COL6A3, COL8A1	20.1284837	7.72E-21	1.62E-19	1.70E-19	1.47E-19
GOTERM_BP_DIRECT	GO:0098609~cell-cell adhesion	14	18.9189189	LAMA5, COL14A1, SRC, ITGA3, ITGA2, LAMA3, ITGA2B, EGFR, ITGAV, ITGA6, TLN2, ITGA5, TLN1, CD44	18.87331947	3.52E-13	2.67E-10	2.96E-11	2.68E-11
GOTERM_BP_DIRECT	GO:0007155~cell adhesion	38	51.3513514	ITGB1, COL18A1, COL15A1, CD151, ITGB4, SRC, ITGB3, PXN, LAMA3, ITGA2B, LAMC2, LAMC1, VTN, ITGAV, AMELX, POSTN, LAMB3, VWF, DST, ITGA3, AMBN, ITGA2, FN1, LAMB1, COL1A1, COL5A1, COL6A2, COL4A3, COL6A1, AMTN, COL6A3, COL8A1, ITGA6, TLN2, ITGA5, CD44, BCAR1, VCL	17.64907841	1.46E-37	1.10E-34	1.10E-34	1.00E-34
KEGG_PATHWAY	hsa04611:Platelet activation	13	17.5675676	ITGB1, VWF, SRC, ITGB3, ITGA2, ITGA2B, GP6, COL1A1, COL3A1, COL1A2, FYN, TLN2, TLN1	17.09860012	5.63E-12	6.47E-10	6.47E-11	4.78E-11
GOTERM_BP_DIRECT	GO:0016477~cell migration	17	22.972973	ITGB1, LAMA5, CD151, LAMB3, ITGB4, ITGB3, PXN, LAMA3, LAMB1, LAMC1, PTK2, VTN, COL5A1, ITGAV, GPC4, CD44, BCAR1	16.07529652	4.99E-15	3.79E-12	4.73E-13	4.28E-13
KEGG_PATHWAY	hsa05165:Human papillomavirus infection	31	41.8918919	ITGB1, LAMA5, ITGB4, ITGB3, PXN, LAMA3, ITGA2B, LAMC2, LAMC1, EGFR, VTN, ITGAV, LAMB3, VWF, ITGA3, ITGA2, FN1, LAMB1, PTK2, COL1A1, COL1A2, COL4A2, COL4A4, COL6A2, COL4A3, COL6A1, COL6A3, GRB2, ITGA6, ITGA5, SOS1	15.27469646	1.04E-29	1.20E-27	4.00E-28	2.96E-28
UP_SEQ_FEATURE	DISULFID:Interchain	10	13.5135135	COL1A1, LAMA5, COL3A1, LAMB3, COL11A1, LAMA3, COL6A3, LAMB1, LAMC2, LAMC1	15.18091862	1.71E-08	1.25E-05	6.59E-07	5.96E-07
UP_KW_BIOLOGICAL_PROCESS	KW-0130~Cell adhesion	34	45.9459459	ITGB1, LAMA5, COL18A1, COL15A1, COL14A1, ITGB4, SRC, ITGB3, PXN, LAMA3, ITGA2B, LAMC2, LAMC1, VTN, ITGAV, POSTN, LAMB3, VWF, DST, ITGA3, ITGA2, FN1, LAMB1, COL6A2, COL4A3, COL6A1, AMTN, COL6A3, COL8A1, ITGA6, ITGA5, CD44, BCAR1, VCL	14.79635654	1.28E-32	2.18E-31	2.18E-31	2.18E-31
KEGG_PATHWAY	hsa05205:Proteoglycans in cancer	18	24.3243243	ITGB1, SRC, LUM, ITGB3, ITGA2, PXN, FN1, EGFR, PTK2, COL1A1, VTN, COL1A2, FLNA, GRB2, ITGAV, ITGA5, SOS1, CD44	14.3204786	1.35E-15	1.53E-13	1.94E-14	1.43E-14
GOTERM_CC_DIRECT	GO:0005925~focal adhesion	22	29.7297297	ITGB1, CD151, DST, ITGB4, SRC, ITGA3, ITGB3, ITGA2, PXN, ITGA2B, EGFR, PTK2, FLNA, ITGAV, ITGA6, TLN2, ITGA5, TLN1, CD44, VCL, BCAR1, PLEC	14.14354216	1.39E-18	2.91E-16	4.85E-17	3.72E-17
KEGG_PATHWAY	hsa04151:PI3K-Akt signaling pathway	30	40.5405405	ITGB1, LAMA5, ITGB4, ITGB3, LAMA3, ITGA2B, LAMC2, LAMC1, EGFR, VTN, ITGAV, LAMB3, VWF, ITGA3, ITGA2, FN1, LAMB1, PTK2, COL1A1, COL1A2, COL4A2, COL4A4, COL6A2, COL4A3, COL6A1, COL6A3, GRB2, ITGA6, ITGA5, SOS1	13.62905345	4.03E-27	4.64E-25	1.16E-25	8.57E-26
KEGG_PATHWAY	hsa04926:Relaxin signaling pathway	10	13.5135135	COL1A1, COL3A1, COL1A2, COL4A2, SRC, COL4A4, COL4A3, GRB2, SOS1, EGFR	12.64297206	5.94E-08	6.83E-06	5.25E-07	3.88E-07
KEGG_PATHWAY	hsa04810:Regulation of actin cytoskeleton	17	22.972973	ITGB1, ITGB4, SRC, ITGA3, ITGB3, ITGA2, PXN, ITGA2B, FN1, EGFR, PTK2, ITGAV, ITGA6, ITGA5, SOS1, VCL, BCAR1	12.10744006	1.55E-13	1.78E-11	1.98E-12	1.47E-12
GOTERM_BP_DIRECT	GO:0030335~positive regulation of cell migration	12	16.2162162	ITGB1, FAM83H, COL1A1, CD151, ITGAV, ITGA6, LAMB1, LAMC2, ITGA5, PTK2, EGFR, BCAR1	11.81475858	4.45E-09	3.37E-06	2.25E-07	2.04E-07
GOTERM_BP_DIRECT	GO:0001525~angiogenesis	10	13.5135135	COL18A1, COL15A1, COL4A2, ITGA2B, FN1, FLNA, COL8A1, ITGAV, ITGA5, PTK2	10.30895601	4.64E-07	3.52E-04	1.76E-05	1.59E-05
UP_KW_CELLULAR_COMPONENT	KW-0965~Cell junction	16	21.6216216	ITGB1, COL17A1, DST, ITGB4, SRC, ITGA3, ITGB3, PXN, PTK2, ITGAV, TLN2, ITGA5, TLN1, VCL, BCAR1, PLEC	9.605062098	6.47E-11	1.29E-09	3.24E-10	2.91E-10
KEGG_PATHWAY	hsa05131:Shigellosis	11	14.8648649	ITGB1, SRC, PXN, TLN2, ITGA5, TLN1, CD44, PTK2, EGFR, VCL, BCAR1	7.263310671	1.66E-06	1.91E-04	1.13E-05	8.32E-06
GOTERM_CC_DIRECT	GO:0009986~cell surface	16	21.6216216	ITGB1, CD151, ITGB4, ITGA3, ITGB3, ITGA2, ITGA2B, GP6, EGFR, ITGAV, ITGA6, ITGA5, GPC4, TLN1, AMELX, CD44	6.794849644	7.79E-09	1.63E-06	1.48E-07	1.13E-07
KEGG_PATHWAY	hsa05200:Pathways in cancer	20	27.027027	ITGB1, LAMA5, LAMB3, ITGA3, ITGA2, LAMA3, ITGA2B, FN1, LAMB1, LAMC2, LAMC1, EGFR, PTK2, COL4A2, COL4A4, COL4A3, GRB2, ITGAV, ITGA6, SOS1	6.14291298	7.91E-11	9.09E-09	8.26E-10	6.11E-10
GOTERM_CC_DIRECT	GO:0005576~extracellular region	43	58.1081081	COL17A1, LAMA5, COL18A1, COL15A1, COL14A1, COL11A1, LAMA3, KLK4, LAMC2, LAMC1, MMP20, VTN, HPX, FLNA, AMELX, COL26A1, LAMB3, VWF, LUM, ODAM, AMBN, FN1, LAMB1, PCOLCE, MMP8, COL1A1, COL3A1, COL1A2, COL4A2, COL5A1, ENAM, COL4A4, COL6A2, COL4A3, COL5A2, COL6A1, AMTN, COL6A3, COL8A1, TLN1, ODAPH, VCL, FBN1	5.433687084	1.45E-22	3.04E-20	6.08E-21	4.66E-21
UP_KW_CELLULAR_COMPONENT	KW-0964~Secreted	46	62.1621622	COL17A1, LAMA5, COL18A1, COL15A1, COL14A1, COL11A1, LAMA3, KLK4, LAMC2, LAMC1, EGFR, MMP20, VTN, HPX, GPC4, AMELX, POSTN, COL26A1, CRTAP, LAMB3, VWF, LUM, ODAM, AMBN, FN1, P3H1, LAMB1, PCOLCE, MMP8, COL1A1, COL3A1, COL1A2, COL4A2, COL5A1, ENAM, COL4A4, COL6A2, COL4A3, COL5A2, COL6A1, AMTN, COL6A3, COL8A1, ODAPH, CD44, FBN1	5.193679893	6.11E-24	1.22E-22	6.11E-23	5.50E-23
UP_KW_LIGAND	KW-0106~Calcium	20	27.027027	ITGB1, FKBP10, DST, ITGB4, ITGA3, ITGB3, COL11A1, ITGA2, ITGA2B, MMP8, MMP20, COL1A1, COL3A1, COL1A2, COL5A1, COL5A2, ITGAV, ITGA6, ITGA5, FBN1	4.909975496	1.07E-10	1.07E-09	1.07E-09	1.07E-09
GOTERM_CC_DIRECT	GO:0005615~extracellular space	35	47.2972973	COL17A1, LAMA5, COL18A1, COL15A1, COL14A1, COL11A1, LAMC2, LAMC1, EGFR, MMP20, VTN, HPX, SERPINH1, POSTN, CRTAP, VWF, LUM, ODAM, FN1, LAMB1, PCOLCE, MMP8, COL1A1, COL3A1, COL1A2, COL4A2, COL5A1, COL4A4, COL6A2, COL4A3, COL5A2, COL6A1, COL6A3, COL8A1, FBN1	4.907272851	3.35E-16	6.96E-14	1.00E-14	7.65E-15
GOTERM_CC_DIRECT	GO:0070062~extracellular exosome	33	44.5945946	ITGB1, LAMA5, COL18A1, COL15A1, ITGB4, SRC, ITGB3, LAMA3, ITGA2B, LAMC1, VTN, HPX, FLNA, ITGAV, GPC4, VWF, ITGA3, LUM, FN1, P3H1, LAMB1, PCOLCE, GP6, COL1A2, COL4A2, COL6A2, COL6A1, COL6A3, GRB2, TLN1, CD44, VCL, PLEC	4.11046694	6.34E-13	1.33E-10	1.47E-11	1.13E-11
GOTERM_CC_DIRECT	GO:0048471~perinuclear region of cytoplasm	11	14.8648649	ITGB1, SRC, ITGA3, ITGA2, FLNA, FYN, LAMB1, LAMC2, PTK2, EGFR, PLEC	4.070488924	3.09E-04	0.06246574	0.00293146	0.00224418
UP_KW_BIOLOGICAL_PROCESS	KW-0945~Host-virus interaction	12	16.2162162	ITGB1, HPX, CD151, SRC, ITGA2, ITGB3, GRB2, ITGAV, FYN, ITGA5, TLN1, EGFR	3.791702559	2.02E-04	0.003428	0.00114451	0.00114451
UP_SEQ_FEATURE	COMPBIAS:Pro residues	23	31.0810811	COL17A1, COL18A1, COL26A1, COL15A1, COL14A1, COL11A1, PTK2, FAM83H, COL1A1, COL3A1, COL1A2, COL4A2, COL5A1, ENAM, COL4A4, COL6A2, COL4A3, COL5A2, COL8A1, SOS1, AMELX, VCL, BCAR1	3.095760004	1.59E-06	0.00115805	3.51E-05	3.18E-05
UP_SEQ_FEATURE	CARBOHYD:N-linked (GlcNAc,,,) asparagine	49	66.2162162	ITGB1, FKBP10, COL18A1, COL14A1, ITGB4, ITGB3, ITGA2B, LAMC2, LAMC1, ITGAV, POSTN, VWF, ITGA3, ITGA2, P3H1, PCOLCE, MMP8, COL4A2, ENAM, COL4A4, COL6A2, COL4A3, COL6A1, COL6A3, ITGA6, ITGA5, CD44, COL17A1, LAMA5, COL15A1, CD151, COL11A1, LAMA3, KLK4, EGFR, VTN, HPX, SERPINH1, GPC4, COL26A1, CRTAP, LAMB3, FN1, LAMB1, GP6, COL1A1, COL1A2, COL5A2, FBN1	3.080500052	5.35E-16	4.06E-13	1.30E-13	1.18E-13
UP_KW_DOMAIN	KW-0732~Signal	58	78.3783784	ITGB1, FKBP10, COL18A1, COL14A1, ITGB4, ITGB3, ITGA2B, LAMC2, LAMC1, MMP20, ITGAV, POSTN, VWF, ITGA3, ITGA2, P3H1, PCOLCE, MMP8, COL4A2, ENAM, COL4A4, COL6A2, COL4A3, COL6A1, AMTN, COL6A3, COL8A1, ITGA6, ITGA5, CD44, LAMA5, COL15A1, COL11A1, LAMA3, KLK4, EGFR, VTN, HPX, SERPINH1, GPC4, AMELX, COL26A1, CRTAP, LAMB3, ODAM, LUM, AMBN, FN1, LAMB1, GP6, COL1A1, COL3A1, COL1A2, COL5A1, COL5A2, ODAPH, BCAR1, FBN1	2.738607062	2.41E-19	4.10E-18	2.05E-18	1.69E-18
GOTERM_CC_DIRECT	GO:0005783~endoplasmic reticulum	10	13.5135135	VTN, FKBP10, CRTAP, COL1A2, VWF, COL4A3, LAMA3, SERPINH1, P3H1, ITGA5	2.374583094	0.02233071	0.99108498	0.08975227	0.06870987
UP_KW_DOMAIN	KW-0677~Repeat	56	75.6756757	ITGB1, FKBP10, COL18A1, COL14A1, ITGB4, ITGB3, ITGA2B, LAMC2, LAMC1, MMP20, ITGAV, POSTN, VWF, DST, ITGA3, ITGA2, P3H1, PCOLCE, WDR72, MMP8, COL4A2, COL4A4, COL6A2, COL4A3, COL6A1, COL6A3, COL8A1, ITGA6, ITGA5, VCL, PLEC, COL17A1, LAMA5, COL15A1, COL11A1, PXN, LAMA3, EGFR, VTN, HPX, FLNA, AMELX, COL26A1, LAMB3, LUM, AMBN, FN1, LAMB1, GP6, COL1A1, COL3A1, COL1A2, COL5A1, COL5A2, GRB2, FBN1	2.310441847	1.86E-14	3.17E-13	1.06E-13	8.70E-14
UP_KW_MOLECULAR_FUNCTION	KW-0675~Receptor	13	17.5675676	ITGB1, ITGB4, ITGA3, ITGB3, ITGA2, ITGA2B, GP6, EGFR, GRB2, ITGAV, ITGA6, ITGA5, CD44	2.301232573	0.00528247	0.12401987	0.02641237	0.02641237
UP_KW_PTM	KW-1015~Disulfide bond	44	59.4594595	COL17A1, ITGB1, LAMA5, COL18A1, COL15A1, COL14A1, ITGB4, COL11A1, ITGB3, LAMA3, ITGA2B, KLK4, LAMC2, LAMC1, EGFR, MMP20, VTN, HPX, ITGAV, POSTN, COL26A1, LAMB3, VWF, ITGA3, LUM, ITGA2, FN1, LAMB1, PCOLCE, GP6, MMP8, COL1A1, COL3A1, COL1A2, COL4A2, COL5A1, COL4A4, COL4A3, COL5A2, COL6A3, ITGA6, ITGA5, CD44, FBN1	2.218532021	6.45E-09	1.35E-07	4.73E-08	4.08E-08
GOTERM_BP_DIRECT	GO:0007165~signal transduction	11	14.8648649	FAM83H, COL15A1, SRC, AMBN, PXN, GRB2, SOS1, AMELX, EGFR, BCAR1, FBN1	2.212442358	0.02365178	0.99999999	0.17238505	0.15623817
UP_KW_PTM	KW-0325~Glycoprotein	54	72.972973	ITGB1, FKBP10, COL18A1, COL14A1, ITGB4, ITGB3, ITGA2B, LAMC2, LAMC1, ITGAV, POSTN, VWF, ITGA3, ITGA2, P3H1, PCOLCE, MMP8, COL4A2, ENAM, COL4A4, COL6A2, COL4A3, COL6A1, AMTN, COL6A3, ITGA6, ITGA5, CD44, COL17A1, LAMA5, COL15A1, CD151, COL11A1, LAMA3, KLK4, EGFR, VTN, HPX, SERPINH1, GPC4, COL26A1, CRTAP, LAMB3, ODAM, LUM, AMBN, FN1, LAMB1, GP6, COL1A1, COL3A1, COL1A2, COL5A2, FBN1	2.196293581	4.59E-12	9.63E-11	5.04E-11	4.36E-11
UP_KW_PTM	KW-0449~Lipoprotein	10	13.5135135	CD151, DST, SRC, ITGA3, ITGB4, FYN, ITGA6, GPC4, EGFR, VCL	2.0481615	0.04992832	0.65889834	0.122047	0.10540423
GOTERM_MF_DIRECT	GO:0046872~metal ion binding	21	28.3783784	ITGB1, COL18A1, POSTN, ITGB4, ITGA3, ITGB3, COL11A1, ITGA2, PXN, ITGA2B, KLK4, COL1A1, HPX, COL3A1, COL1A2, COL5A1, COL5A2, ITGAV, FYN, ITGA6, ITGA5	2.031598115	0.00185598	0.25158808	0.01378727	0.01042883
GOTERM_MF_DIRECT	GO:0042802~identical protein binding	13	17.5675676	COL18A1, VWF, ITGB3, ITGA2B, FN1, EGFR, COL1A1, VTN, COL1A2, GRB2, FYN, AMELX, FBN1	1.972694252	0.02676688	0.98548445	0.12281274	0.09289681
UP_KW_CELLULAR_COMPONENT	KW-0966~Cell projection	10	13.5135135	ITGB1, DST, ITGA3, ITGB3, FLNA, TLN1, CD44, PTK2, VCL, BCAR1	1.945292732	0.06512925	0.73996525	0.26051702	0.23446532
GOTERM_CC_DIRECT	GO:0005886~plasma membrane	29	39.1891892	COL17A1, ITGB1, CD151, ITGB4, SRC, ITGB3, PXN, ITGA2B, EGFR, FLNA, ITGAV, FYN, GPC4, COL26A1, ITGA3, ITGA2, FN1, GP6, PTK2, GRB2, ITGA6, TLN2, ITGA5, TLN1, SOS1, CD44, BCAR1, VCL, PLEC	1.483298239	0.01691826	0.97173605	0.07216155	0.05524329
UP_KW_LIGAND	KW-0479~Metal-binding	25	33.7837838	ITGB1, FKBP10, COL18A1, ITGB4, COL11A1, ITGB3, PXN, ITGA2B, KLK4, MMP20, HPX, ITGAV, FYN, DST, ITGA3, ITGA2, P3H1, MMP8, COL1A1, COL3A1, COL1A2, COL5A1, COL5A2, ITGA6, ITGA5	1.4769474	0.00186934	0.01853694	0.00934671	0.00934671
UP_SEQ_FEATURE	REGION:Disordered	62	83.7837838	ITGB1, FKBP10, COL18A1, COL14A1, ITGB4, ITGB3, ITGA2B, LAMC2, LAMC1, MMP20, ITGAV, POSTN, DST, ITGA3, P3H1, PCOLCE, WDR72, MMP8, COL4A2, ENAM, COL4A4, COL6A2, COL4A3, COL6A1, AMTN, COL6A3, COL8A1, ITGA5, SOS1, CD44, VCL, PLEC, COL17A1, LAMA5, COL15A1, CD151, SRC, COL11A1, PXN, LAMA3, EGFR, FAM83H, VTN, HPX, FLNA, FYN, AMELX, COL26A1, ODAM, AMBN, FN1, LAMB1, GP6, PTK2, COL1A1, COL3A1, COL1A2, COL5A1, COL5A2, ODAPH, BCAR1, FBN1	1.253677143	0.00107118	0.54317484	0.01168709	0.01056794
GOTERM_MF_DIRECT	GO:0005515~protein binding	59	79.7297297	ITGB1, FKBP10, ITGB4, ITGB3, ITGA2B, MMP20, ITGAV, POSTN, VWF, DST, ITGA3, ITGA2, P3H1, PCOLCE, COL4A2, ENAM, COL6A2, COL4A3, AMTN, COL8A1, ITGA6, TLN2, ITGA5, TLN1, SOS1, CD44, VCL, PLEC, COL17A1, CD151, SRC, PXN, KLK4, EGFR, FAM83H, VTN, HPX, SERPINH1, FLNA, FYN, GPC4, AMELX, CRTAP, LAMB3, ODAM, LUM, AMBN, FN1, LAMB1, GP6, PTK2, COL1A1, COL3A1, COL1A2, COL5A1, GRB2, ODAPH, BCAR1, FBN1	1.227968932	0.00388486	0.45513573	0.0275472	0.02083698

Supplemental Table 3. KEGG pathways on DAVID. *All pathways and "go terms" that contained at least 10 of the genes identified by the previous analysis were included in Database for Annotation, Visualization and Integrated Discovery (DAVID) and they have been classified by enrichment score on this table.*

Statistical analysis

Statistical analysis was performed using pair-wise Anova test and post-hoc Tukey's test. Statistical significance level was considered as $p < 0.05$. Data were analyzed using PRISM 6.0 (GraphPad, La Jolla, CA, USA). All experiments have been performed at least three times from three different cell culture batches (biological and technical replicates).

On SEM microscopy images, one-way analysis of variance (ANOVA) was assessed using the Sigma plot software (release 11.2, Systat Software, Inc., San Jose, CA, USA) to determine whether significant differences existed in the mass percentage of Ca. In all tests, a statistical significance level of $\alpha = 0.05$ was adopted.

Acknowledgments

Authors thank first all family individuals participating to this study and Pr Marie-Paule GELLE (DDS) from the Competence Centre for Rare Oral and Dental Diseases O-Rares of Reims and Dr Jean-Christophe DAHLET (DDS) and Dr Delphine WAGNER (DDS, orthodontic specialist) as part of the O-Rares network and who participated to some consultations of affected individuals. Authors also thank Dr Maria Del Mar Muniz Moreno for her help on the interactome conception on Cytoscape™. Authors acknowledge Dr. Karen NIEDERREITHER and Dr. Patrick REILLY for her help on the manuscript construction and English language review. The authors declare non-financial conflicts of interest related to this study.

Financial support

This work was financed by and contributed to the actions of the projects Offensives Sciences INTERREG IV A27 and "RARENET: No. 1.7, a trinational network for education, research and management of complex and rare disorders in the Upper Rhine" co-financed by the European Regional Development Fund (ERDF) of the European Union in the framework of the INTERREG V Upper Rhine program as well as to the ERN (European reference network) CRANIO initiative. ABZ is a USIAS 2015 Fellow of the Institute of Advanced Studies (Institut d'Etudes Avancées) de l'Université de Strasbourg, France. It was also supported by the grant ANR-10-LABX-0030-INRT, a French State fund managed by the Agence Nationale de la Recherche under the frame programme Investissements d'Avenir labelled ANR-10-IDEX-0002-02. This work is also part of the Projet E-GENODENT financed by the Fonds d'Intervention Régionale (FIR) of the Agence Régionale de Santé Grand Est (2019-2022). The authors thank the "Impulsion Recherche" financial support of the "Filière de Santé Maladies Rares TETECOU" 2021 and 2022.

The funders had no role in the study design, data collection and analysis, decision to publish, or preparation of the manuscr

All references

1. Goertz O, Baerreiter S, Ring A, Jettkant B, Hirsch T, Daigeler A, et al. Determination of microcirculatory changes and angiogenesis in a model of frostbite injury in vivo. J Surg Res. 2011 Jun 1;168(1):155–61.
2. Bourne MH, Piepkorn MW, Clayton F, Leonard LG. Analysis of microvascular changes in frostbite injury. J Surg Res. 1986 Jan;40(1):26–35.
3. Rey T, Tarabeux J, Gerard B, Delbarre M, Le Béchec A, Stoetzel C, et al. Protocol GenoDENT: Implementation of a New NGS Panel for Molecular Diagnosis of Genetic Disorders with Orodental Involvement. Methods Mol Biol. 2019;1922:407–52.
4. Prasad MK, Geoffroy V, Vicaire S, Jost B, Dumas M, Le Gras S, et al. A targeted next-generation sequencing assay for the molecular diagnosis of genetic disorders with orodental involvement. J Med Genet. 2016 Feb;53(2):98–110.
5. Lisignoli G, Lambertini E, Manferdini C, Gabusi E, Penolazzi L, Paolella F, et al. Collagen type XV and the 'osteogenic status'. J Cell Mol Med. 2017 Sep;21(9):2236–44.
6. Wu J, Ren W, Zheng Z, Huang Z, Liang T, Li F, et al. Mmu_circ_003795 regulates osteoblast differentiation and mineralization in MC3T3-E1 and MDPC23 by targeting COL15A1. Mol Med Rep. 2020 Sep;22(3):1737–46.
7. Chen KL, Yeh YY, Lung J, Yang YC, Yuan K. Mineralization Effect of Hyaluronan on Dental Pulp Cells via CD44. J Endod. 2016 May;42(5):711–6.
8. Wang X, Jin T, Chang S, Zhang Z, Czajka-Jakubowska A, Nör JE, et al. In Vitro Differentiation and Mineralization of Dental Pulp Stem Cells on Enamel-Like Fluorapatite Surfaces. Tissue Eng Part C Methods. 2012 Nov [cited 2022 Sep 30];18(11):821–30.
9. Rasi K, Piuhola J, Czabanka M, Sormunen R, Ilves M, Leskinen H, et al. Collagen XV is necessary for modeling of the extracellular matrix and its deficiency predisposes to cardiomyopathy. Circ Res. 2010 Nov 12;107(10):1241–52.
10. Eklund L, Piuhola J, Komulainen J, Sormunen R, Ongvarrasopone C, Fássler R, et al. Lack of type XV collagen causes a skeletal myopathy and cardiovascular defects in mice. Proc Natl Acad Sci U S A. 2001 Jan 30;98(3):1194–9.
11. Guillon E, Bretaud S, Ruggiero F. Slow Muscle Precursors Lay Down a Collagen XV Matrix Fingerprint to Guide Motor Axon Navigation. J Neurosci. 2016 Mar 2;36(9):2663–76.
12. Paolella F, Gabusi E, Manferdini C, Schiavinato A, Lisignoli G. Specific concentration of hyaluronan amide derivative induces osteogenic mineralization of human mesenchymal stromal cells: Evidence of RUNX2 and COL1A1 genes modulation. J Biomed Mater Res A. 2019 Dec;107(12):2774–83.
13. Kim J, Lee G, Chang WS, Ki S hyoung, Park JC. Comparison and Contrast of Bone and Dentin in Genetic Disorder, Morphology and Regeneration: A Review. J Bone Metab. 2021 Feb;28(1):1–10.
14. Gatseva A, Sin YY, Brezzo G, Van Agtmael T. Basement membrane collagens and disease mechanisms. Essays Biochem. 2019 Sep 13;63(3):297–312.

15. McGrath J. The structure and function of skin – ClinicalKey. BOOK CHAPTER. McKee's Pathology of the Skin, Chapter 1, 1-34.e3 44.
16. Myllyharju J, Kivirikko KI. Collagens and collagen-related diseases. Ann Med. 2001 Feb;33(1):7–21.
17. Gensemer C, Burks R, Kautz S, Judge DP, Lavallee M, Norris RA. Hypermobile Ehlers-Danlos syndromes: Complex phenotypes, challenging diagnoses, and poorly understood causes. Developmental Dynamics. 2021 [cited 2022 Nov 2];250(3):318–44.
18. Grahame R. Joint hypermobility and genetic collagen disorders: are they related? Archives of Disease in Childhood. 1999 Feb 1 [cited 2022 Nov 2];80(2):188–91.
19. Steiner RD, Basel D. COL1A1/2 Osteogenesis Imperfecta. In: Adam MP, Ardinger HH, Pagon RA, Wallace SE, Bean LJ, Gripp KW, et al., editors. GeneReviews®. Seattle (WA): University of Washington, Seattle; 1993.
20. Valadares ER, Carneiro TB, Santos PM, Oliveira AC, Zabel B. What is new in genetics and osteogenesis imperfecta classification? J Pediatr (Rio J). 2014 Dec;90(6):536–41.
21. Chen KL, Yeh YY, Lung J, Yang YC, Yuan K. Mineralization Effect of Hyaluronan on Dental Pulp Cells via CD44. J Endod. 2016 May;42(5):711–6.
22. Ibrahim S, Strange AP, Aguayo S, Shinawi A, Harith N, Mohamed-Ibrahim N, et al. Phenotypic Properties of Collagen in Dentinogenesis Imperfecta Associated with Osteogenesis Imperfecta. Int J Nanomedicine. 2019 Dec 2 [cited 2022 Sep 29];14:9423–35.
23. Khalilgharibi N, Mao Y. To form and function: on the role of basement membrane mechanics in tissue development, homeostasis and disease. Open Biol. 2021 Feb;11(2):200360.
24. Pozzi A, Yurchenco PD, Iozzo RV. The nature and biology of basement membranes. Matrix Biol. 2017 Jan [cited 2022 Nov 2];57–58:1–11.
25. Rosenberg RJ, Schilder H. The basement membrane of the enamel organ in human odontogenesis. Oral Surgery, Oral Medicine, Oral Pathology. 1984 May 1 [cited 2020 Apr 16];57(5):544–53.
26. Kruegel J, Miosge N. Basement membrane components are key players in specialized extracellular matrices. Cell Mol Life Sci. 2010 Sep 1 [cited 2022 Nov 2];67(17):2879–95.
27. Sawada T, Inoue S. Ultrastructural verification of anchoring role of lamina fibroreticularis of dental basement membrane in odontogenesis. J Electron Microsc (Tokyo). 1999 Jan 1 [cited 2020 Apr 16];48(6):919–28.
28. Heljasvaara R, Aikio M, Ruotsalainen H, Pihlajaniemi T. Collagen XVIII in tissue homeostasis and dysregulation - Lessons learned from model organisms and human patients. Matrix Biol. 2017 Jan;57–58:55–75.
29. Mutolo MJ, Morris KJ, Leir SH, Caffrey TC, Lewandowska MA, Hollingsworth MA, et al. Tumor suppression by collagen XV is independent of the restin domain. Matrix Biol. 2012 Jun;31(5):285–9.
30. Exome Aggregation Consortium, Lek M, Karczewski KJ, Minikel EV, Samocha KE, Banks E, et al. Analysis of protein-coding genetic variation in 60,706 humans. Nature. 2016 Aug;536(7616):285–91.
31. Bloch-Zupan A, Rousseaux M, Laugel V, Schmittbuhl M, Mathis R, Desforges E, et al. A possible cranio-oro-facial phenotype in Cockayne syndrome. Orphanet J Rare Dis. 2013 Jan 14;8:9.

32. Li H, Durbin R. Fast and accurate long-read alignment with Burrows–Wheeler transform. Bioinformatics. 2010 Mar 1;26(5):589–95.
33. The 1000 Genomes Project Consortium, Gibbs RA, Boerwinkle E, Doddapaneni H, Han Y, Korchina V, et al. A global reference for human genetic variation. Nature. 2015 Oct;526(7571):68–74.
34. Geoffroy V, Herenger Y, Kress A, Stoetzel C, Piton A, Dollfus H, et al. AnnotSV: An integrated tool for Structural Variations annotation. Bioinformatics. 2018 Apr 14;
35. Yeo G, Burge CB. Maximum entropy modeling of short sequence motifs with applications to RNA splicing signals. J Comput Biol. 2004;11(2–3):377–94.
36. Reese MG, Eeckman FH, Kulp D, Haussler D. Improved splice site detection in Genie. J Comput Biol. 1997;4(3):311–23.
37. Shapiro MB, Senapathy P. Laboratory of Statistical and Mathematical Methodology, Division of Computer Research and Technology, National Institutes of Health, Bethesda, MD 20892, USA. Nucleic Acids Research. :20.
38. Laugel-Haushalter V, Bär S, Schaefer E, Stoetzel C, Geoffroy V, Alembik Y, et al. A New SLC10A7 Homozygous Missense Mutation Responsible for a Milder Phenotype of Skeletal Dysplasia With Amelogenesis Imperfecta. Front Genet. 2019;10:504.
