# Supplementary material for: Rare dentin defects: Understanding the pathophysiological mechanisms of COLXVA1 mutations
Source: Genes Dis. 2024 Apr 20;11(5):101303. doi: 10.1016/j.gendis.2024.101303 (PMC11074959; doi:10.1016/j.gendis.2024.101303)
Supplement: Multimedia component 2 [file mmc2.pdf]

Gene	gNomen	cNomen	pNomen
COL15A1	Chr9(GRCh37):g.101798451G>C	NM_001855.4:c.2290-1G>C	p.?
ECI2/C6orf201	Chr6(GRCh37):g.4126439G>C	NM_001166010.1:c.514C>G	p.Leu172Val
DNAJC10	Chr2(GRCh37):g.183622522C>A	NM_018981.2:c.1913C>A	p.Pro638His
C7orf50	Chr7(GRCh37):g.1049689G>A	NM_001318252.1:c.220C>T	p.Arg74Trp
PLXDC2	Chr10(GRCh37):g.20357145G>A	NM_001282736.1:c.371G>A	p.Arg124His
CCDC174	Chr3(GRCh37):g.14695975T>A	NM_016474.4:c.85T>A	p.Phe29Ile
COL3A1	Chr2(GRCh37):g.189875017A>C	NM_000090.3:c.3937A>C	p.Lys1313Gln
TTN/-AS1	Chr2(GRCh37):g.179425550C>T	NM_001267550.1:c.85309G>A	p.Gly28437Ser
EML3	Chr11(GRCh37):g.62376557C>T	NM_153265.2:c.806G>A	p.Arg269His
FAM135A	Chr6(GRCh37):g.71234640T>C	NM_001162529.1:c.1853T>C	p.Leu618Pro
CD248	Chr11(GRCh37):g.66082338G>A	NM_020404.2:c.2161C>T	p.Arg721Cys
CSAD	Chr12(GRCh37):g.53566174A>T	NM_015989.4:c.381T>A	p.His127Gln
VPS13A	Chr9(GRCh37):g.79931168C>T	NM_033305.2:c.4709C>T	p.Thr1570Ile
LRIG2	Chr1(GRCh37):g.113637017C>T	NM_001312686.1:c.263C>T	p.Ser88Leu
SPTA1	Chr1(GRCh37):g.158612638C>A	NM_003126.2:c.4571G>T	p.Cys1524Phe
MTOR	Chr1(GRCh37):g.11189856C>T	NM_004958.3:c.5653G>A	p.Val1885Ile
CPZ	Chr4(GRCh37):g.8607794A>G	NM_003652.3:c.755A>G	p.Gln252Arg
TDRD3	Chr13(GRCh37):g.61059936T>C	NM_001146071.1:c.292T>C	p.Cys98Arg
TFB2M	Chr1(GRCh37):g.246707851G>A	NM_022366.2:c.991C>T	p.Arg331Cys
PTPRC	Chr1(GRCh37):g.198721796A>C	NM_002838.4:c.3404A>C	p.Lys1135Thr
LDB3	Chr10(GRCh37):g.88441535G>A	NM_001171610.1:c.664G>A	p.Ala222Thr
KLHDC8A	Chr1(GRCh37):g.205307683G>A	NM_001271863.1:c.799C>T	p.Arg267Trp
RBMXL2	Chr11(GRCh37):g.7111255T>C	NM_014469.4:c.904T>C	p.Tyr302His
SERINC4/SERF2	Chr15(GRCh37):g.44089343T>C	NM_001258031.1:c.844A>G	p.Lys282Glu
WBSCR22	Chr7(GRCh37):g.73100975G>A	NM_001202560.2:c.96G>A	p.Met32Ile
MLLT10	Chr10(GRCh37):g.21823619C>T	NM_004641.3:c.46C>T	p.His16Tyr
FLNC	Chr7(GRCh37):g.128485216C>A	NM_001458.4:c.3697C>A	p.His1233Asn
KCTD8	Chr4(GRCh37):g.44177114T>C	NM_198353.2:c.1115A>G	p.Asn372Ser
TMEM245	Chr9(GRCh37):g.111819564C>A	NM_032012.3:c.1761G>T	p.Leu587Phe
CLK2	Chr1(GRCh37):g.155240714G>T	NM_001294338.1:c.55C>A	p.Arg19Ser
TM9SF4	Chr20(GRCh37):g.30730905G>C	NM_014742.3:c.649G>C	p.Glu217Gln
TRIM66	Chr11(GRCh37):g.8642663T>C	NM_014818.1:c.2933A>G	p.Asn978Ser
CCDC168	Chr13(GRCh37):g.103385998C>G	NM_001146197.1:c.17049G>C	p.Lys5683Asn
CCDC88C	Chr14(GRCh37):g.91780445G>A	NM_001080414.3:c.1715C>T	p.Ser572Leu
POLE2	Chr14(GRCh37):g.50117111A>G	NM_002692.3:c.1369T>C	p.Tyr457His
ROBO4	Chr11(GRCh37):g.124757104G>A	NM_019055.5:c.2204C>T	p.Pro735Leu
FGF4	Chr11(GRCh37):g.69588175G>A	NM_002007.2:c.523C>T	p.Pro175Ser
MRVI1/-AS1	Chr11(GRCh37):g.10597900A>T	NM_130385.3:c.2718T>A	p.His906Gln
AKAP13	Chr15(GRCh37):g.86225419A>G	NM_006738.5:c.5144A>G	p.Asn1715Ser
ANKEF1/SNAP25-AS1	Chr20(GRCh37):g.10030280A>G	NM_022096.5:c.1063A>G	p.Ser355Gly
CBS/L	Chr21(GRCh37):g.44474003C>T	NM_001321072.1:c.1328G>A	p.Arg443Gln
C1QTNF6	Chr22(GRCh37):g.37581353G>C	NM_031910.3:c.194C>G	p.Pro65Arg
CCDC70	Chr13(GRCh37):g.52439779G>A	NM_031290.2:c.265G>A	p.Glu89Lys
SLCO6A1	Chr5(GRCh37):g.101816007A>G	NM_173488.4:c.490T>C	p.Phe164Leu
C3orf20	Chr3(GRCh37):g.14768516A>G	NM_032137.4:c.1675A>G	p.Ile559Val
CDK10	Chr16(GRCh37):g.89753128C>G	NM_052988.4:c.10C>G	p.Pro4Ala
F13A1	Chr6(GRCh37):g.6175022A>T	NM_000129.3:c.1538T>A	p.Met513Lys
MSLNL	Chr16(GRCh37):g.830094T>C	NM_001025190.1:c.907A>G	p.Lys303Glu
ARHGEF12	Chr11(GRCh37):g.120352130A>G	NM_015313.2:c.4399A>G	p.Ile1467Val
SYT8	Chr11(GRCh37):g.1857428G>A	NM_001290332.1:c.475G>A	p.Val159Ile
SPATC1	Chr8(GRCh37):g.145095307G>A	NM_198572.2:c.709G>A	p.Gly237Arg
HERPUD1	Chr16(GRCh37):g.56969215G>T	NM_014685.3:c.216G>T	p.Leu72Phe
ERRFI1	Chr1(GRCh37):g.8073686A>C	NM_018948.3:c.973T>G	p.Leu325Val
