# Supplementary material for: Rare dentin defects: Understanding the pathophysiological mechanisms of COLXVA1 mutations
Source: Genes Dis. 2024 Apr 20;11(5):101303. doi: 10.1016/j.gendis.2024.101303 (PMC11074959; doi:10.1016/j.gendis.2024.101303)
Supplement: Multimedia component 3 [file mmc3.pdf]

Gene	Forward primer	Reverse primer
COL1A1	5'-TGGGAGTGCAAGGATACTCTATATCG-3'	5'-CCCATCCCATCTTCGACGTAC-3'
COL15A1	5'-GGTGACACTGGTTTACCTGGCT-3'	5'-GCCTTTCCAGAGGAATGTCCTC-3'
COL3A	5'-CTGGACCAAAAGGTGATGCT-3'	5'-CAGGGTTTCCATCTCTTCCA-3'
DSPP	5'-CCTAAAGAAAATGAAGATAATT-3'	5'-TAGAAAAACTCTTCCCTCCTAC-3'
GADPH	5'-CCACCCATGGCAAATTCCATGGCA-3'	5'-TCTAGACGGCAGGTCAGGTCCACC-3'
MMP20	5'-TCCATCCCTGACCTCTGTGACT-3'	5'-AGTGAACCTGCCGTCTCCAGAA-3'

Target	Characteristics	Reference
**Ameloblastin**	mouse monoclonal anti-AMBN	sc-271012, Santa Cruz
**Ameloblastin**	rabbit monoclonal anti-AMBN	sc-50534, Santa Cruz
**Amelogenin**	mouse monoclonal anti-AMELX	sc-365284, Santa Cruz
**Enamelin**	goat polyclonal anti-ENAM	sc-33107, Santa Cruz
**COL1A1**	mouse monoclonal anti-COL1A1	sc-293182, Santa Cruz
**MMP20**	rabbit polyclonal anti-MMP20	bs-5788R, Bioss
**FAM83H**	rabbit polyclonal anti-FAM83H	sc-PA5-55094, Thermofisher
**Goat IgG**	Donkey Fluor™ 488	A-11055, Thermofisher
**Rabbit IgG**	Donkey Fluor™ 488	A-21206, Thermofisher
**Mouse IgG**	Donkey Fluor™ 488	A-21202, Thermofisher
**Goat IgG**	Donkey Fluor™ 594	A-11058, Thermofisher
**Rabbit IgG**	Donkey Fluor™ 546	A10040, Thermofisher
**Mouse IgG**	Donkey Fluor™ 568	A10037, Thermofisher
